# Tolerability, toxicity, and outcomes following surgical and non-surgical approaches to the management of patients with locally advanced oesophageal squamous cell carcinoma: multicentre retrospective cohort study

**DOI:** 10.1093/bjsopen/zraf078

**Published:** 2025-09-08

**Authors:** The RadioRoux Collaborative, A Cockbain, Aaron Kisiel, Aisha Anwer, Ajay Mehta, Alex Boddy, Amy Irwin, Ashley Poon-King, Ashwin Krishnamoorthy, Atia Khan, Ben Grace, Betsan Thomas, C Jones, Charles Rayner, Claire Livings, Hospital Trust, Craig Barrington, Daniel Otter, Daniel Newport, David Fackrell, Devesh Kaushal, David McIntosh, E A Griffiths, Ellhia Sudin, Emma Upchurch, Elena Theophilidou, Fiona Crotty, G Radhakrishna, George Mori, Geta Maharaj, Gillian Miller, Gwen Edwards, Haider Abbas, Harris Siddique, Helen Lada, Iain Wilson, Ioannis Sarantitis, J Bundred, J Gossage, J Sultan, J A Elliott, Jane Dunn, John Reynolds, Jonathan Helbrow, Jonathan Lessing, Joy Murphy, Judith Sayers, Julie Walther, Kaveetha Kandiah, Katarina Chow, Hospital Trust, Kathryn Hogan, Katie Roulston, Khalid Akbari, Kish Pursnani, L Brown, Lewis Gall, Lewis Germain, Liam Hyland, Laura McGuinness, Marie Kershaw, Mashal Ahmed, Matthaios Kapiris, Matthew Doe, Mayar Aswad, Mohammed Hamid, Mohammed Hoque, Mohan Hingorani, Michael Glaysher, Michael Hughes, Nakul Viswanath, N Blencowe, N W Han, Nathalie Webber, Nicholas Penney, Nicholas Maynard, Nicholas Gomez, Nila Tewari, Nitya Matcha, Hospital Trust, Nia Jackson, Paul Williams, P Charlton, P Coe, P Pucher, Hospital Trust, P Singh, Poppy Jones, R Samuel, R Goody, R Owen, Raj Nijjar, Rania Mohammed, Reem Mahmood, R P T Evans, Rob Walker, S Markar, Said Alyacoubi, Saleem Noormohamed, Sara Walker, Sarah Gwynne, Shameen Jaunoo, Shiv Uppal, Sinthuri Raveendran, Siobhan Chien, Siona Growcott, S K Kamarajah, Sufyan Azam, Swetha Prathap, T Crosby, T J Underwood, Tasia Aghadiuno, Terence Lo, Waleed Al-Khyatt, William Knight, Hospital Trust, Y M Goh, Zubair Khanzada

## Abstract

**Background:**

Oesophageal squamous cell carcinoma is the predominant histopathological subtype of oesophageal cancer across the world, representing as many as 90% of all cases; however, within Western cohorts, it is a low-prevalence disease, and, as such, appropriately powered trials to establish a standard treatment paradigm in this population remain challenging. The aim of this study was to assess current practices and compare outcomes for patients with locally advanced oesophageal squamous cell carcinoma across the UK and Ireland.

**Methods:**

This was a retrospective multicentre cohort study of patients managed with curative intent for squamous cell carcinoma of the middle or distal oesophagus in 23 hospitals across the UK and Ireland. Consecutive patients diagnosed between 1 January 2012 and 31 December 2016 were included.

**Results:**

This study included 1545 patients, of whom 923 (59.7%) received definitive chemoradiotherapy, 286 (18.5%) neoadjuvant chemotherapy + surgery, 218 (14.1%) neoadjuvant chemoradiotherapy + surgery, and 118 (7.6%) surgery alone. Neoadjuvant chemoradiotherapy + surgery was associated with significantly longer survival than neoadjuvant chemotherapy or definitive chemoradiotherapy (median 83.9 *versus* 27.8 *versus* 26.5 months). In propensity score-matched analysis of overall survival, patients receiving neoadjuvant chemoradiotherapy + surgery had significantly longer survival than those who had definitive chemoradiotherapy (median 56.8 *versus* 43.1 months; hazard ratio 0.39, 95% confidence interval 0.20 to 0.78; *P* < 0.001).

**Conclusion:**

This multicentre retrospective cohort study suggests that, despite a majority of patients being treated with definitive chemoradiotherapy, patients undergoing neoadjuvant chemoradiotherapy and surgery have improved survival compared with those receiving definitive chemoradiotherapy or neoadjuvant chemotherapy + surgery. In the absence of robust Western randomized clinical trial data, neoadjuvant chemoradiotherapy + surgery should be considered the standard for well selected patients fit for surgery.

## Teaser

This multicentre cohort study analysed survival outcomes of patients managed with curative intent for oesophageal squamous cell carcinoma. Patients undergoing neoadjuvant chemoradiotherapy and surgery had improved survival compared with patients receiving definitive chemoradiotherapy or neoadjuvant chemotherapy + surgery. In the absence of robust Western randomized clinical trial data, neoadjuvant chemoradiotherapy + surgery should be considered the standard for well selected, fit patients.

## Introduction

Oesophageal cancer is a leading cause of cancer-related morbidity and mortality globally, contributing to the loss of around 9.8 million disability-adjusted life-years and 436 000 deaths each year^1^. Oesophageal squamous cell carcinoma (OSCC) is the predominant histopathological subtype across much of the world, representing as many as 85.0% of all oesophageal cancers^2^. In contrast, OSCC accounts for a comparatively lower proportion of all oesophageal malignancies in Western practice, including in the UK, which has an age-standardized rate of 2.1 per 100 000 person-years, translating to approximately 3000 new diagnoses annually^3^.

A number of treatment options exist for patients diagnosed with locally advanced OSCC, including definitive chemoradiotherapy (dCRT) and either neoadjuvant chemotherapy (nCT) or neoadjuvant chemoradiotherapy (nCRT) followed by planned surgical resection^4–6^. Since publication of the Checkmate 577 trial^7^, patients with stage II and III oesophageal and gastro-oesophageal junctional tumours for whom nCRT does not achieve a pathological complete response have additionally benefit from adjuvant immune checkpoint inhibition. In addition, there is increasing interest in the use of upfront dCRT with salvage surgery as required, an approach that is currently under evaluation in the multicentre NEEDS (NCT04460352) trial^8^.

Although there is strong evidence that nCT and nCRT improve survival *versus* surgery alone, their efficacy relative to one another and to dCRT is uncertain^9^. Consequently, these approaches are treated with equipoise within European and UK guidance, and there remain limited data to guide clinicians in selecting and counselling for each specific treatment approach^10^.

The authors sought to compare survival, tolerability, and toxicity outcomes following dCRT and surgery preceded by nCRT or nCT in a large, multicentre cohort study of patients with locally advanced OSCC managed within the UK and Ireland.

## Methods

### Ethics and governance

Each participating centre received relevant approvals from their local National Health Service Research and Development departments, and data were collected by members of the care team. Each study subject was link anonymized, and no identifiable data were shared outside of contributing centres.

### Study design and reporting

This was a retrospective, multicentre cohort study incorporating patients from the UK and Ireland. Collation of routinely collected data was led by the Roux Group, the trainee arm of the Association of Upper Gastrointestinal Surgeons of Great Britain and Ireland (AUGIS), and the UK National Oncology Trainees Collaborative for Healthcare Research (NOTCH)^11^. This report was prepared in accordance with STROBE^12^ and STROCSS^13^ guidance.

### Setting

Data were collected from eligible British and Irish centres. All included centres offer surgical resection and have access to centralized radiotherapy services. Patients from centres unable to deliver radiotherapy were discussed at central upper gastrointestinal multidisciplinary team (MDT) meetings and referred for radiotherapy at alternative centres, all of which participated in this study.

### Patient population

Consecutive patients aged at least 18 years recently diagnosed with OSCC affecting the middle- or lower-third of the oesophagus between 1 January 2012 and 31 December 2016 were included, allowing for a minimum of 3 years of follow-up after completion of treatment. Patients with an upper-third or cervical OSCC (defined as within 20 cm of the incisors), those with non-squamous histology, and patients who had previously received treatment for an oesophageal cancer were excluded. Tumours were otherwise defined as middle- or lower-third lesions based on endoscopic assessment. Decisions relating to treatment were made by a specialist upper gastrointestinal MDT at each site, in accordance with local and national guidance^14^. Only patients for whom a potentially curative treatment approach was selected by the MDT were included in this analysis. Patients with tumour category T4 disease were included if they were treated with curative intent. Patients who were identified as having potentially curative disease, but because of limited performance status or personal choice did not proceed to treatment with curative intent, were not included. Patients undergoing only endoscopic therapy were excluded because of absence of clinical comparability to the wider population.

### Data collection

Relevant patients were identified using one or more paper and electronic health records, radiotherapy treatment databases, chemotherapy treatment databases, upper gastrointestinal cancer MDT meeting records, and individual surgical treatment records. Contributing centres were able to choose which of these would most robustly provide a consecutive series of patients for inclusion in this study. Data were subsequently collected by trained medical investigators at each participating centre by inputting into a prepiloted database hosted on the REDCap® version 11.0 secure web application (Vanderbilt University, Nashville, TN). All inputted data were reviewed centrally and queries resolved with the submitting team before analysis.

Data items extracted related to patient demographics, tumour characteristics, diagnostic and pretreatment work-up, and treatment, in addition to treatment toxicity and tolerability, as well as survival outcomes. Performance status was classified by Eastern Cooperative Oncology Group (ECOG) criteria. Extent of co-morbidity was assessed using the Charlson Co-morbidity Index (CCI). Tumour characteristics were drawn from pathology reports, and measurements relating to tumour length and location from endoscopic assessment or, where this was not available, from imaging reports. Two-stage surgery included either open or minimally invasive surgery, in both abdominal and thoracic phases. Three-stage surgery was defined by the addition of a cervical phase. A positive pathological margin was defined by the presence of tumour < 1 mm from the surgical margin, in accordance with guidance from the Royal College of Pathologists^15^. Extracted radiotherapy data related to the planned and given dose, the number of fractions over which treatment was delivered, and the total duration of treatment. Data relating to chemotherapy included the dose, number of cycles delivered, and whether there were interruptions to treatment or dose adjustments. Surgical data included the type of procedure and extent of lymphadenectomy. Complications relating to nCT or nCRT were defined retrospectively in accordance with Common Terminology Criteria for Adverse Events^16^. Complications relating to surgery were graded according to the Clavien–Dindo classification^17^. Patient follow-up was based on local procedures. Overall follow-up time and status at last known contact or visit were also recorded.

### Sample size

All relevant UK and Irish MDTs managing patients with OSCC were invited to participate and provide data on consecutive patients. No formal sample size calculation was undertaken.

### Statistical analysis

Categorical variables were compared using the χ^2^ test, and non-normally distributed data using the Mann–Whitney *U* test. Survival was estimated using Kaplan–Meier survival curves and compared by means of the log rank test. Cox proportional hazards models were used for multivariable analyses. *P* < 0.050 was considered statistically significant. Data analysis was undertaken using R version 3.2.2 (R Foundation for Statistical Computing, Vienna, Austria) with the TableOne, ggplot2, Hmisc, Matchit, and survival packages.

The conditional probability of survival (that is, the propensity score) was estimated using a multivariable logistic regression model. The following baseline co-variables were included in the model: age, sex, ECOG performance status, CCI score, tumour grade, tumour location, tumour length, staging modality, and stage as defined by American Joint Committee on Cancer criteria. Clinical stage and variables collected after surgical resection of cancer were used, as these represent the available data that clinicians use to make decisions regarding neoadjuvant treatment. A propensity score was calculated using the nearest-neighbour method, with a calliper width equal to 0.1 standard deviation

## Results

### All patients

#### Cohort and treatment approach

Overall, 1545 consecutive patients were included from 23 participating British and Irish centres (England, 18; Scotland, 1; Wales, 2; Ireland, 2) (*supplementary methods*). Reflecting considerable intercentre variation in treatment approach, the proportion of patients managed using dCRT within each centre ranged from 10.5 to 100% (*Fig. S1*); nevertheless, a majority of patients were scheduled for (915, 59.2%), or actually received (923, 59.7%) dCRT. Although the MDT planned nCT + surgery for 232 patients (15.0%), and nCRT + surgery for 307 (19.8%), nCT + surgery was started in 286 (18.5%) and nCRT + surgery in 218 (14.1%).

In contrast to patients for whom dCRT was planned, those with a primary MDT treatment plan inclusive of surgery demonstrated significant deviations in their treatment plan: 26.7% of patients assigned to receive nCT + surgery or nCRT + surgery did not have an operation. Deviation from planned surgery arose because of reduction in performance status, or progression of disease or patient choice. The number of patients assigned to receive surgery alone was 98 (6.3%); however, 118 (7.6%) underwent surgery alone.

#### Baseline characteristics

Baseline patient characteristics are presented in *Table 1*. Patients receiving dCRT were older, had more co-morbidities, and a higher frequency of middle-third cancers than those receiving nCRT or nCT. Furthermore, patients receiving dCRT were more likely to have locally advanced T3/T4a disease. Length of tumour did not differ in patients receiving nCRT + surgery (median 5.0 (interquartile range 4.0–6.0) cm) and nCT + surgery (5.0 (4.0–6.0) cm). Patients undergoing surgery alone had significantly earlier disease stages, with high proportions of T1 (55, 46%) and N0 (95, 80.5%) disease. In addition to gastroscopy, computed tomography (CT) (1448, 93.7%) and positron emission tomography–CT (1459, 94.4%) were the most frequently used staging modalities. Others, including endoscopic ultrasonography (995, 64.4%), magnetic resonance imaging (58, 3.8%), and endobronchial ultrasonography (45, 2.9%) were used less frequently.

**Table 1:** Baseline characteristics of patients with oesophageal squamous cell carcinoma by treatment intent

		dCRT (*n*= 923)	nCRT (*n*= 218)	nCT (*n*= 286)	Surgery only (*n*= 118)	Total (*n*= 1545)	P*
**Age (years)**							< 0.001
18–44		19 (2.1%)	8 (3.7%)	2 (0.7%)	2 (1.7%)	31 (2.0%)	
45–59		151 (16.4%)	68 (31.2%)	74 (25.9%)	23 (19.5%)	316 (20.5%)	
60–79		638 (69.1%)	142 (65.1%)	200 (69.9%)	72 (61.0%)	1052 (68.1%)	
≥ 80		108 (11.7%)	0 (0%)	8 (2.8%)	21 (17.8%)	137 (8.9%)	
Missing		7 (0.8%)	0 (0%)	2 (0.7%)	0 (0%)	9 (0.6%)	
**Sex**							0.772
Male		379 (41.1%)	93 (42.7%)	128 (44.8%)	51 (43.2%)	651 (42.1%)	
Female		525 (56.9%)	123 (56.4%)	154 (53.8%)	66 (55.9%)	868 (56.2%)	
Missing		19 (2.1%)	2 (0.9%)	4 (1.4%)	1 (0.8%)	26 (1.7%)	
**ECOG status**							< 0.001
0		339 (36.7%)	133 (61.0%)	135 (47.2%)	54 (45.8%)	661 (42.8%)	
1		443 (48.0%)	76 (34.9%)	115 (40.2%)	49 (41.5%)	683 (44.2%)	
≥ 2		141 (15.3%)	9 (4.1%)	36 (12.6%)	15 (12.7%)	201 (13.0%)	
**CCI score**							0.004
0		587 (63.6%)	161 (73.9%)	216 (75.5%)	72 (61.0%)	1036 (67.1%)	
1–2		241 (26.1%)	41 (18.8%)	49 (17.1%)	33 (28.0%)	364 (23.6%)	
≥3		21 (2.3%)	2 (0.9%)	5 (1.7%)	2 (1.7%)	30 (1.9%)	
Missing		74 (8.0%)	14 (6.4%)	16 (5.6%)	11 (9.3%)	115 (7.4%)	
**Tumour grade (differentiation)**							< 0.001
Well		53 (5.7%)	11 (5.0%)	24 (8.4%)	17 (14.4%)	105 (6.8%)	
Moderate		422 (45.7%)	98 (45.0%)	133 (46.5%)	45 (38.1%)	698 (45.2%)	
Poor		222 (24.1%)	60 (27.5%)	87 (30.4%)	25 (21.2%)	394 (25.5%)	
Unknown		226 (24.5%)	49 (22.5%)	42 (14.7%)	31 (26.3%)	348 (22.5%)	
**Basaloid-type SCC**							0.107
No		688 (74.5%)	147 (67.4%)	222 (77.6%)	81 (68.6%)	1138 (73.7%)	
Yes		35 (3.8%)	8 (3.7%)	8 (2.8%)	3 (2.5%)	54 (3.5%)	
Unknown		200 (21.7%)	63 (28.9%)	56 (19.6%)	34 (28.8%)	353 (22.8%)	
**Tumour location**							< 0.001
Middle third		506 (54.8%)	86 (39.4%)	82 (28.7%)	50 (42.4%)	724 (46.9%)	
Lower third		366 (39.7%)	110 (50.5%)	176 (61.5%)	51 (43.2%)	703 (45.5%)	
GOJ		25 (2.7%)	13 (6.0%)	23 (8.0%)	9 (7.6%)	70 (4.5%)	
Unknown		26 (2.8%)	9 (4.1%)	5 (1.7%)	8 (6.8%)	48 (3.1%)	
Tumour length (cm), median (i.q.r.)		4.5 (3.0–6.0)	5.0 (4.0–6.0)	5.0 (4.0–6.0)	3.0 (2.0–4.2)	4.7 (3.0–6.0)	< 0.001†
**Staging CT**							< 0.001
No		42 (4.6%)	23 (10.6%)	9 (3.1%)	23 (19.5%)	97 (6.3%)	
Yes		881 (95.4%)	195 (89.4%)	277 (96.9%)	95 (80.5%)	1448 (93.7%)	
**Staging PET**							< 0.001
No		46 (5.0%)	10 (4.6%)	6 (2.1%)	24 (20.3%)	86 (5.6%)	
Yes		877 (95.0%)	208 (95.4%)	280 (97.9%)	94 (79.7%)	1459 (94.4%)	
**MRI**							0.259
No		887 (96.1%)	211 (96.8%)	272 (95.1%)	117 (99.2%)	1487 (96.2%)	
Yes		36 (3.9%)	7 (3.2%)	14 (4.9%)	1 (0.8%)	58 (3.8%)	
**Staging EUS**							0.190
No		344 (37.3%)	72 (33.0%)	101 (35.3%)	33 (28.0%)	550 (35.6%)	
Yes		579 (62.7%)	146 (67.0%)	185 (64.7%)	85 (72.0%)	995 (64.4%)	
**Staging EBUS**							0.272
No		900 (97.5%)	209 (95.9%)	279 (97.6%)	112 (94.9%)	1500 (97.1%)	
Yes		23 (2.5%)	9 (4.1%)	7 (2.4%)	6 (5.1%)	45 (2.9%)	
**Staging laparoscopy**							< 0.001
No		856 (92.7%)	178 (81.7%)	177 (61.9%)	90 (76.3%)	1301 (84.2%)	
Yes		67 (7.3%)	40 (18.3%)	109 (38.1%)	28 (23.7%)	244 (15.8%)	
**AJCC clinical tumour category**							< 0.001
cT1a		15 (1.6%)	1 (0.5%)	1 (0.3%)	25 (21.2%)	42 (2.7%)	
cT1b		55 (6.0%)	7 (3.2%)	9 (3.1%)	30 (25.4%)	101 (6.5%)	
cT2		139 (15.1%)	33 (15.1%)	40 (14.0%)	35 (29.7%)	247 (16.0%)	
cT3		601 (65.1%)	167 (76.6%)	216 (75.5%)	26 (22.0%)	1010 (65.4%)	
cT4a		94 (10.2%)	9 (4.1%)	18 (6.3%)	2 (1.7%)	123 (8.0%)	
cT4b		19 (2.1%)	1 (0.5%)	2 (0.7%)	0 (0%)	22 (1.4%)	
**AJCC clinical node category**							< 0.001
cN0		395 (42.8%)	74 (33.9%)	91 (31.8%)	95 (80.5%)	655 (42.4%)	
cN1		404 (43.8%)	106 (48.6%)	140 (49.0%)	16 (13.6%)	666 (43.1%)	
cN2		116 (12.6%)	34 (15.6%)	42 (14.7%)	7 (5.9%)	199 (12.9%)	
cN3		8 (0.9%)	4 (1.8%)	13 (4.5%)	0 (0%)	25 (1.6%)	
**Surgery**							< 0.001
No		879 (95.2%)	47 (21.6%)	82 (28.7%)	0 (0%)	1008 (65.2%)	
Yes		44 (4.8%)	171 (78.4%)	204 (71.3%)	118 (100%)	537 (34.8%)	

Values are *n* (%) unless otherwise stated. dCRT, definitive chemoradiotherapy; nCRT, neoadjuvant chemoradiotherapy; nCT, neoadjuvant chemotherapy; ECOG, Eastern Cooperative Oncology Group; CCI, Charlson Co-morbidity Index; SCC, squamous cell carcinoma; GOJ, gastro-oesophageal junction; i.q.r., interquartile range; CT, computed tomography; PET, positron emission tomography; MRI, magnetic resonance imaging; EUS, endoscopic ultrasonography; EBUS, endobronchial ultrasonography; AJCC, American Joint Committee on Cancer. *χ^2^ test, except †Mann–Whitney *U* test.

#### Treatment toxicity: non-surgical

Grade 3 haematological toxicity rates did not differ significantly between dCRT (5.68%), nCRT (6.81%), and nCT (5.23%) groups (*Table S1*). Patients managed with dCRT had significantly higher rates of non-haematological complications (29.3%) compared with those who received nCRT (20.1%; *P* = 0.002) or nCT (18.6%; *P* = 0.001). Underlying this, although uncommon across cohorts, electrolyte derangement was significantly more common with dCRT (2.7%) than nCRT (0.9%; *P* = 0.03) or nCT (0.3%; *P* = 0.011). Similarly, pulmonary toxicity was encountered more often with dCRT (6.2%) than nCRT (5.1%; *P* = 0.012) or nCT (2.0%; *P* = 0.031). Interestingly, a greater proportion of patients receiving nCRT (2.98%) reported grade 3 weight loss compared with those receiving dCRT (1.4%; *P* = 0.04) or nCT (0.65%; *P* = 0.036).

#### Overall survival

**Fig. 1:**
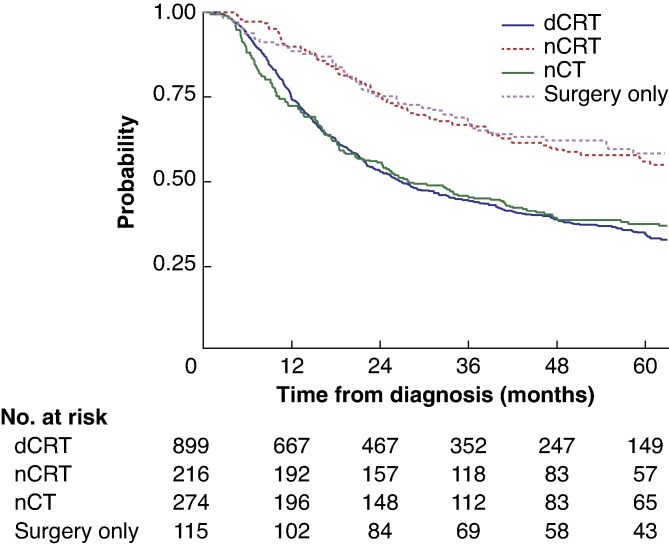
Overall survival of intention-to-treat cohorts Time- from diagnosis. dCRT, definitive chemoradiotherapy; nCRT, neoadjuvant chemoradiotherapy; nCT, neoadjuvant chemotherapy. *P* < 0.001 (log rank test).

Patients assigned to nCRT followed by surgery had significantly longer survival than those receiving nCT or dCRT (median 83.9 *versus* 27.8 *versus* 26.5 months) (*Fig. 1*). In adjusted multivariable analysis for overall survival, patients receiving nCRT had significantly longer survival than those who had dCRT (hazard ratio (HR) 0.51, 95% confidence interval (c.i.) 0.36 to 0.72; *P* < 0.001); however, there was no significant difference in survival between those receiving only nCT or surgery compared with dCRT (*Table 2*).

**Table 2:** Adjusted Cox regression analysis of long-term survival of patients with SCC

	Univariable analysis		Multivariable analysis	
	Hazard ratio	*P*	Hazard ratio	*P*
**Age (years)**				
18–44	1.00 (reference)		1.00 (reference)	
45–59	1.04 (0.42, 2.59)	0.929	1.87 (0.42, 8.34)	0.409
60–79	0.97 (0.40, 2.36)	0.945	1.87 (0.43, 8.08)	0.400
≥ 80	1.29 (0.47, 3.55)	0.622	2.08 (0.35, 12.34)	0.420
**Sex**				
Male	1.00 (reference)		1.00 (reference)	
Female	0.65 (0.51, 0.85)	0.001	0.47 (0.32, 0.70)	< 0.001
Unknown	1.05 (0.33, 3.29)	0.940	1.34 (0.17, 10.35)	0.782
**ECOG status**				
0	1.00 (reference)		1.00 (reference)	
1	1.58 (1.21, 2.06)	0.001	1.15 (0.76, 1.72)	0.516
≥ 2	1.38 (0.88, 2.17)	0.164	1.21 (0.64, 2.29)	0.561
**CCI score**				
0	1.00 (reference)		1.00 (reference)	
1–2	1.16 (0.84, 1.60)	0.361	1.30 (0.82, 2.07)	0.270
≥3	1.05 (0.34, 3.29)	0.934	0.00 (0.00, Inf)	0.993
**Tumour grade (differentiation)**				
Well	1.00 (reference)		1.00 (reference)	
Moderate	2.10 (1.13, 3.91)	0.019	1.87 (0.82, 4.23)	0.135
Poor	2.32 (1.23, 4.38)	0.010	1.68 (0.73, 3.86)	0.221
Unknown	1.96 (1.02, 3.77)	0.045	0.86 (0.31, 2.45)	0.783
**Basaloid-type SCC**				
No	1.00 (reference)		1.00 (reference)	
Yes	0.57 (0.25, 1.28)	0.171	0.31 (0.07, 1.30)	0.108
Unknown	0.99 (0.74, 1.32)	0.929	1.04 (0.63, 1.72)	0.872
**Tumour location**				
Middle third	1.00 (reference)		1.00 (reference)	
Lower third	0.90 (0.68, 1.18)	0.438	0.56 (0.37, 0.85)	0.006
GOJ	1.05 (0.64, 1.75)	0.837	0.67 (0.34, 1.33)	0.252
Unknown	0.99 (0.48, 2.04)	0.984	0.46 (0.05, 4.37)	0.499
**Tumour length (cm)**				
1	1.02 (0.96, 1.09)	0.440	1.01 (0.94, 1.09)	0.759
1.5 to < 4	1.30 (1.24, 1.21)	0.040	1.10 (1.05, 2.17)	0.046
4 to < 6	2.37 (1.38, 4.27)	0.029	1.62 (0.65, 7.42)	0.326
≥ 6	3.17 (1.19, 8.25)	0.022	1.94 (0.49, 8.80)	0.327
**AJCC clinical tumour category**				
cT1a	1.00 (reference)		1.00 (reference)	
cT1b	1.24 (0.50, 3.03)	0.642	0.21 (0.03, 1.37)	0.103
cT2	1.45 (0.64, 3.28)	0.368	1.19 (0.33, 4.35)	0.792
cT3	2.48 (1.16, 5.28)	0.019	1.73 (0.48, 6.32)	0.405
cT4a	2.77 (1.09, 7.05)	0.032	1.74 (0.39, 7.80)	0.467
cT4b	–		–	
**AJCC clinical node category**				
cN0	1.00 (reference)		1.00 (reference)	
cN1	0.99 (0.75, 1.31)	0.965	0.83 (0.54, 1.28)	0.404
cN2	1.31 (0.89, 1.92)	0.178	0.87 (0.47, 1.59)	0.642
cN3	1.41 (0.52, 3.83)	0.501	2.27 (0.43, 12.03)	0.335
**AJCC clinical metastasis category**				
cM0	1.00 (reference)		1.00 (reference)	
cM1	6.29 (0.87, 45.22)	0.068	3.06 (0.33, 28.28)	0.324
**Final treatment**				
dCRT	1.00 (reference)		1.00 (reference)	
nCRT	0.52 (0.33, 0.84)	0.007	0.39 (0.20, 0.78)	0.008
nCT	0.84 (0.54, 1.30)	0.424	0.82 (0.45, 1.49)	0.519
Surgery only	0.59 (0.36, 0.95)	0.032	0.87 (0.39, 1.94)	0.735

Values in parentheses are 95% confidence intervals. SCC, squamous cell carcinoma; ECOG, Eastern Cooperative Oncology Group; CCI, Charlson Co-morbidity Index; GOJ, gastro-oesophageal junction; AJCC, American Joint Committee on Cancer; dCRT, definitive chemoradiotherapy; nCRT, neoadjuvant chemoradiotherapy; nCT, neoadjuvant chemotherapy.

### Cohort after oesophagectomy: dCRT ± salvage surgery, nCT ± surgery, nCRT ± surgery

#### Baseline characteristics

Of 1545 patients, 537 (35%) received surgery following either dCRT (salvage surgery) (44 of 923, 4.8%), nCRT (171 of 218, 78.4%), or nCT (204 of 286, 71.3%), or had surgery alone (118, 7.6%). Baseline characteristics of patients are presented in *Table S2*. A 2-stage operation was undertaken in 366 patients (68.2%), and 3-stage surgery in 156 (29.0%). A 3-field lymphadenectomy was undertaken in 21 patients (3.9%). Patients receiving dCRT and salvage surgery were younger, had lower co-morbidity rates, and had fewer middle-third cancers compared with those receiving nCRT or nCT.

#### Treatment toxicity: surgical

For all patients undergoing surgery, the overall postoperative complication rate was 50.1% (269 patients), and the major complication rate was 26.4% (142 patients) (*Table S3*). The anastomotic leak rate was 11.0% (type 1: 4.7% (25 patients); type 2: 2.4% (13); type 3: 3.9% (21)) and the conduit necrosis rate was 3.5% (19 patients). Pneumonia was the most common complication, affecting 118 patients (22%).

#### Overall survival

**Fig. 2:**
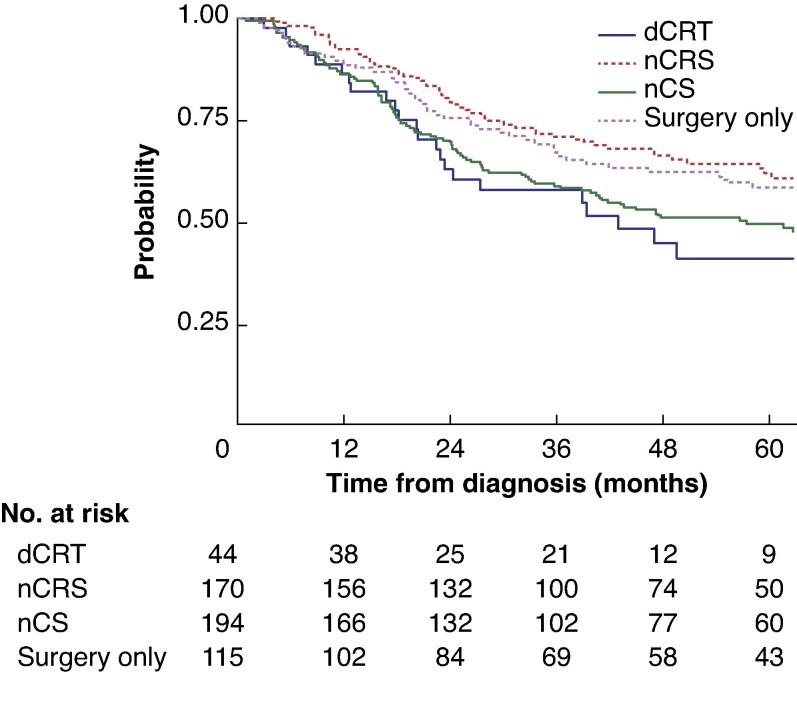
Overall survival after surgery dCRT+S, definitive chemoradiotherapy + surgery; nCRT+S, neoadjuvant chemoradiotherapy + surgery; nCT+S, neoadjuvant chemotherapy + surgery. *P* = 0.004 (log rank test).

Patients receiving nCRT + surgery had significantly longer survival than those receiving surgery after nCT or dCRT (median not reached *versus* 56.8 *versus* 43.1 months; *P* < 0.001) (*Fig. 2*). In adjusted analysis of overall survival, patients receiving nCRT + surgery had significantly longer survival than those receiving dCRT and surgery (HR 0.39, 95% c.i. 0.20 to 0.78; *P* < 0.001); however, there were no significant differences in survival between those receiving nCT and surgery, or surgery only, compared with those who had dCRT and surgery (*Table S4*).

### Propensity score-matched analysis: dCRT *versus* nCRT ± surgery

Patients undergoing dCRT and nCRT + surgery subsequently underwent propensity score matching to further identify an optimum treatment strategy. Patients were matched for patient factors, staging technique, and tumour factors. A total of 581 patients were included: 394 who had undergone dCRT, and 187 who had had nCRT + surgery (*Table S5*).

**Fig. 3:**
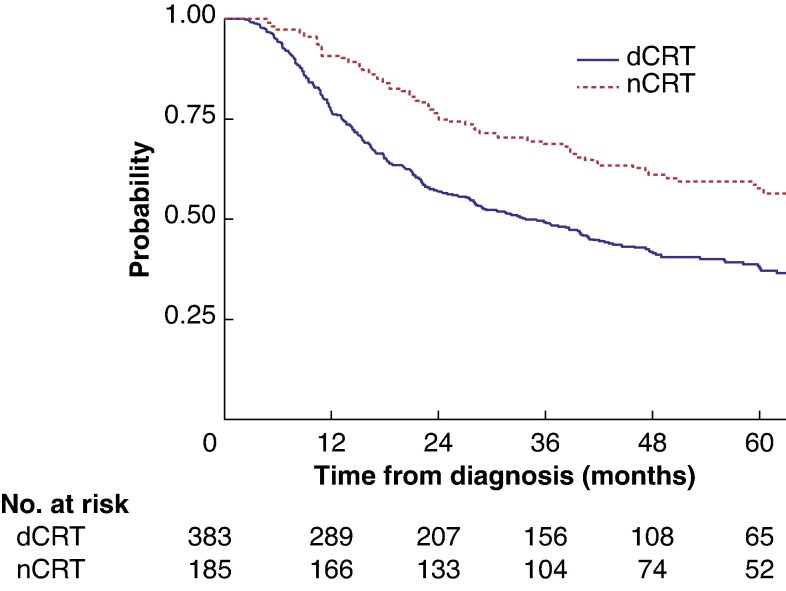
Propensity score-matched survival for patients undergoing dCRT or nCRT + surgery dCRT, definitive chemoradiotherapy; nCRT, neoadjuvant chemoradiotherapy. *P* < 0.001 (log rank test).

Patients receiving nCRT and surgery had significantly longer survival than those receiving dCRT (median 56.8 *versus* 43.1 months; HR 0.39, 95% c.i. 0.20 to 0.78; *P* < 0.001) (*Fig. 3* and *Table 3*).

**Table 3:** Adjusted Cox regression analysis of long-term survival of propensity-matched patients with SCC undergoing dCRT *versus* nCRT + surgery

	No. of patients	Hazard ratio*	*P*
**Age (years)**			
18–44	15 (2.6%)	1.00 (reference)	
45–59	146 (25.1%)	1.21 (0.59, 2.51)	0.599
60–79	420 (72.3%)	1.19 (0.59, 2.41)	0.627
**Sex**			
Female	340 (58.5%)	1.00 (reference)	
Male	236 (40.6%)	1.19 (0.95, 1.48)	0.127
Unknown	5 (0.9%)	0.00 (0.00, ∞)	0.992
**ECOG status**			
0	318 (54.7%)	0.45 (0.30, 0.68)	< 0.001
1	225 (38.7%)	0.62 (0.41, 0.93)	0.021
≥ 2	38 (6.5%)	1.00 (reference)	
**CCI score**			
0	411 (70.7%)	0.72 (0.30, 1.74)	0.462
1–2	126 (21.7%)	0.84 (0.34, 2.07)	0.700
≥ 3	7 (1.2%)	1.00 (reference)	
Missing	37 (6.4%)	0.98 (0.37, 2.59)	0.975
**Tumour grade**			
Moderate	277 (47.7%)	1.00 (reference)	
Poor	148 (25.5%)	0.83 (0.64, 1.09)	0.185
Unknown	128 (22.0%)	0.86 (0.65, 1.14)	0.297
Well	28 (4.8%)	0.59 (0.32, 1.08)	0.086
**Basaloid-type SCC**			
No	415 (71.4%)	1.00 (reference)	
Unknown	144 (24.8%)	1.16 (0.91, 1.50)	0.231
Yes	22 (3.8%)	0.46 (0.22, 0.98)	0.045
**Tumour location**			
GOJ	27 (4.6%)	1.00 (reference)	
Lower third	268 (46.1%)	1.00 (0.58, 1.73)	0.998
Middle third	268 (46.1%)	1.18 (0.68, 2.04)	0.549
Unknown	18 (3.1%)	1.49 (0.69, 3.22)	0.315
**Tumour length (cm)**			
1	13 (2.2)	1.00 (reference)	
1.5 to < 4	78 (13.4)	2.01 (1.10, 33.10)	0.026
4 to < 6	151 (26.0)	2.48 (0.72, 9.96)	0.182
≥ 6	107 (18.3)	2.78 (1.31, 22.17)	0.041
Missing	232 (39.9)	2.35 (0.87, 6.37)	0.092
**AJCC clinical tumour category**			
cT1a	4 (0.7)	1.00 (reference)	
cT1b	22 (3.8)	1.99 (0.25, 15.75)	0.513
cT2	89 (15.3)	1.94 (0.27, 14.11)	0.515
cT3	429 (73.8)	3.21 (0.45, 22.91)	0.244
cT4a	34 (5.9)	5.00 (0.68, 36.99)	0.115
cT4b	3 (0.5)	6.50 (0.68, 62.57)	0.105
**AJCC clinical node category**			
cN0	205 (35.3)	-	
cN1	283 (48.7)	1.07 (0.84, 1.36)	0.610
cN2	85 (14.6)	1.42 (1.03, 1.96)	0.034
cN3	8 (1.4)	1.06 (0.43, 2.59)	0.905
**AJCC clinical metastasis category**			
cM0	578 (99.5)	1.00 (reference)	
cM1	3 (0.5)	1.71 (0.42, 6.86)	0.452
**Surgery**			
None	413 (71.1)	1.00 (reference)	
Yes	168 (28.9)	0.43 (0.33, 0.57)	< 0.001
**Final treatment**			
dCRT	394 (67.8)	1.00 (reference)	
nCRT + surgery	187 (32.2)	0.52 (0.41, 0.67)	< 0.001

*Values in parentheses are 95% confidence intervals. SCC, squamous cell carcinoma; dCRT, definitive chemoradiotherapy; nCRT, neoadjuvant chemoradiotherapy; ECOG, Eastern Cooperative Oncology Group; CCI, Charlson Co-morbidity Index; GOJ, gastro-oesophageal junction; AJCC, American Joint Committee on Cancer.

## Discussion

At present, there is little to inform patients and clinicians when selecting between dCRT and surgical approaches for the management of OSCC^9^. In this large, retrospective analysis, the authors have provided detailed information relating to relative toxicity, tolerability, and outcomes data for surgical and non-surgical treatment approaches used for 1545 patients with potentially curative mid- and lower-third OSCC. The have also provided an insight into trends in practice over a period before the introduction of adjuvant immune checkpoint inhibition for patients managed with nCRT.

Overall, most patients received dCRT; this cohort was older, had increased co-morbidity, and reduced performance status compared with those who received surgery following nCT or nCRT. Interestingly, only 55% of those considered suitable for nCRT + surgery on the basis of MDT discussion subsequently proceeded to trimodal treatment; the remainder were instead selected for dCRT, with the number of patients initially planned for dCRT at the MDT meeting increasing by 14%. This is likely to reflect clinician assessment of fitness for surgery following MDT discussions. The rate of postoperative complications was high in this study; this potentially reflects the high burden of co-morbidity in patients with squamous cell carcinoma compared with adenocarcinoma. In particular, conduit necrosis rates were nearly double those of recent studies^18^.

This provides considerable evidence for case selection, against which the outcomes data presented here must be caveated; however, in propensity score-matched analysis, overall survival was greater in the group that received nCRT + surgery than in those who had dCRT.

There is a relatively low incidence of potentially curative OSCC in Western countries, which has limited the potential for high-quality comparative trials^19^ against which these outcomes data may be compared. Historical data drawn from Stahl *et al.*^20^ and Bedenne *et al.*^21^, both published in the 2000s, demonstrated no significant difference in overall survival for dCRT and nCRT + surgery; however, these studies used historical radiotherapy techniques, and their relevance has been further questioned because of high rates of treatment-related morbidity and issues relating to trial design. Survival outcomes in these studies were also in accordance with contemporary standards, with reported 2-year overall survival rates of 33.6–39.9%, contrasting poorly with those for nCRT + surgery in the CROSS trial^22^, which exceeded 70%. Difficulty in interpreting these data is clearly shown by the wide variation in treatment practices across the 23 included centres in the present study. The authors’ data are, however, in keeping with a recent meta-analysis^23^ incorporating 10 studies comprising 12 132 patients, which reported that patients who received nCRT + surgery had significantly longer survival than those who received dCRT (HR 0.68, 95% c.i. 0.54 to 0.87; *P* < 0.001).

With two treatment modalities showing similar survival outcomes, more information relating to relative treatment-related morbidity may guide patients and clinicians when making treatment choices. In this cohort, 35.0% of patients receiving dCRT experienced a grade 3 or greater complication compared with 26.9% of patients receiving nCRT. Major complications of surgery (Clavien–Dindo grade ≥ III) occurred in 26.4% of patients, similar to rates reported in recent observational series^24^. These data are limited to a short-term analysis of toxicity; however, evidence from the European multicentre LASER study^25^, which evaluated health-related quality of life (HRQoL) outcomes in 876 patients over 1 year after oesophagectomy, determined that two-thirds had ongoing symptoms that were attributable to surgery. Conversely, HRQoL recovered by 6 months after dCRT in the phase II/III SCOPE 1 trial^26^. This potentially worse survivorship after oesophagectomy is advocated as a key benefit of upfront dCRT^10^. The preSANO study^27^ was developed in light of this, and sought to evaluate whether patients for whom nCRT delivered a complete pathological response might proceed to surveillance with delayed oesophagectomy as required. The study led to the SANO trial^28^, a phase III, non-inferiority, stepped-wedge, cluster-randomized clinical trial in which patients with no evidence of residual disease in two consecutive clinical response evaluations after nCRT had either active surveillance or standard oesophagectomy. Data from this study^29^ suggest that patients who underwent active surveillance following nCRT had non-inferior survival outcomes and improved HRQoL compared with those who proceeded to surgery. This contrasts with data presented in the DICE study^30^, which reported that an increased duration of > 200 days after chemoradiotherapy (CRT) increases both 90-day mortality and reduces 5-year survival, further supporting upfront surgery after CRT. The role of active surveillance in patients with a complete response after nCRT or dCRT is yet to be determined. This study did not include patients undergoing surveillance after nCRT; however, survival was improved for patients undergoing nCRT + surgery compared with dCRT + surgery.

This study has also demonstrated that radiotherapy-based management is superior to nCT, with 3-year survival rates of 40.9 and 54.6% for nCT + surgery and nCRT + surgery, respectively. Although these data are drawn from a Western population, they are in keeping with randomized data from China in which, at 1 year after treatment, the rate of death from tumour progression or recurrence was significantly higher following nCT than nCRT^31^. Nevertheless, systemic therapy remains the neoadjuvant standard of care in Asia, and recent meta-analyses^32,33^ have failed to demonstrate a significant difference in survival between nCT and nCRT in comparisons of different international populations; however, new evidence has recently emerged from Japan demonstrating improved outcomes with neoadjuvant triplet chemotherapy^34^. JCOG1109 NExT has failed to demonstrate improved survival with the addition of radiotherapy to doublet chemotherapy (cisplatin, fluorouracil), but did shown survival improvements with the addition of docetaxel. The role of improved systemic therapies in the neoadjuvant setting are poorly understood. Advancing knowledge from Asian cohorts will better guide optimum treatment strategies for Western OSCC. It is likely that, since the study period outlined here, most UK centres will have moved to more frequent use of nCRT in place of nCT. Contributing to this, immune checkpoint inhibition is licensed and funded for patients for whom nCRT does not result in a pathological complete response. This is not accounted for in the data here, which predate the CheckMate 577 trial, the impact of which would likely be a widening of the efficacy gap between nCRT + surgery and dCRT^7^.

A further limitation to this work is that it reflects a retrospective cohort with solely observational data ascertainment. Data points were kept to a minimum to ensure high-quality data acquisition; as such, specific details on CRT were not available. Bias was minimized by the use of outcomes relating to overall survival, which is well captured across different health records, by inclusion of all patients who met inclusion criteria within each centre, regardless of treatment approach. This study is also open to treatment selection bias, as is evidenced by the wide variation in centre-specific treatment practices. Furthermore, within centres, surgeons aim to operate only on suitable candidates; however, propensity score matching provided a mechanism by which the authors attempted to limit such bias. This study also benefits from a large number of patients and a long follow-up of at least 3 years.

**Fig. 4:**
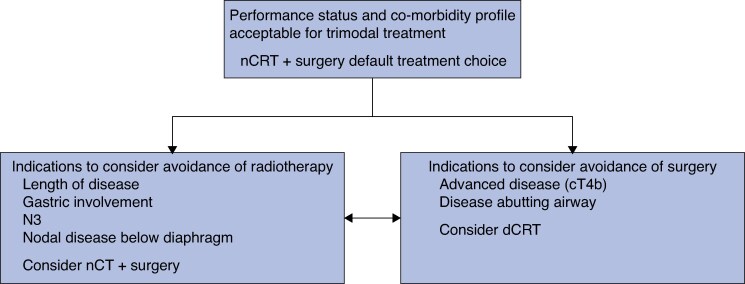
Treatment decision aids for patients with locally advanced squamous cell carcinoma of the mid/lower oesophagus nCRT, neoadjuvant chemoradiotherapy; nCT, neoadjuvant chemotherapy; dCRT, definitive chemoradiotherapy.

Overall, this multicentre retrospective analysis of patients undergoing potentially curative treatment for OSCC has provided evidence that nCRT + surgery can be regarded as the optimum treatment approach for patients with locally advanced OSCC (*Fig. 4*). To evaluate the standard treatment for OSCC in greater detail, further randomized data are required. These will be provided, at least in part, by the multicentre NEEDS trial, which will compare nCRT + surgery for OSCC *versus* dCRT with salvage surgery as needed. This pragmatic trial will allow the use of immunotherapy according to the approval status of the drug and clinical practice regulations in each country. It is yet to be determined whether the intensive 3-monthly surveillance strategy will identify patients suitable for salvage and improve overall survival in the dCRT arm, or whether the morbidity of dCRT will prevent further treatment. Furthermore, the present propensity score-matched analysis has demonstrated that patients receiving nCRT + surgery have significantly better overall survival than those receiving dCRT. Patients undergoing salvage surgery had perioperative morbidity similar to that of patients who had oesophagectomy after neoadjuvant treatment. It is important to recognize that case selection plays an integral role in achieving an optimum survival strategy for patients with potentially curative OSCC; however, within such constraints, and in the absence of high-quality randomized data, this study has provided evidence that nCRT + surgery should be actively considered for fit patients with potentially curative OSCC of the mid/lower oesophagus.

## Collaborators

A. Cockbain, Imperial College Healthcare Trust, London, UK; Aaron Kisiel, Queen Elizabeth Hospital, Birmingham, UK; Aisha Anwer, Heart of England Hospital Trust, Birmingham, UK; Ajay Mehta, Leeds University Hospitals, Leeds, UK; Alex Boddy, University Hospitals Leicester, Leicester, UK; Amy Irwin, Queen Elizabeth Hospital, Birmingham, UK, Annabel Lyles, Leeds University Hospitals, Leeds, UK; Ashley Poon-King, Morriston Hospital, Morriston, UK; Ashwin Krishnamoorthy, University Hospital of Coventry and Warwick, Coventry, UK; Atia Khan, Bradford Royal Infirmary, Bradford, UK; Ben Grace, University Hospital of Southampton, Southampton, UK; Betsan Thomas, University Hospital of Wales, Cardiff, UK; C. Jones, Leeds University Hospitals, Leeds, UK and Cambridge University Hospitals, Cambridge, UK; Charles Rayner, Royal Surrey Hospital, Guildford, UK; Claire Livings, Guy's and St Thomas' Hospital Trust, London, UK; Craig Barrington, University Hospital of Southampton, Southampton, UK; Daniel Otter, Leeds University Hospitals, Leeds, UK; Daniel Newport, University Hospital of Coventry and Warwick, Coventry, UK; David Fackrell, Queen Elizabeth Hospital Birmingham, Birmingham, UK; Devesh Kaushal, University Hospital of Coventry and Warwick, Coventry, UK; David McIntosh, Glasgow Royal Infirmary, Glasgow, UK; E.A. Griffiths, Queen Elizabeth Hospital Birmingham, Birmingham, UK; Ellhia Sudin, University Hospitals Nottingham, Nottingham, UK; Emma Upchurch, University Hospitals Bristol, Bristol, UK; Elena Theophilidou, University Hospitals Nottingham, Nottingham, UK; Fiona Crotty, St James Hospital, Leeds, UK; G. Radhakrishna, Manchester University NHS Trust, Manchester, UK; George Mori, Bradford Royal Infirmary, Bradford, UK; Geta Maharaj, Morriston Hospital, Morriston, UK; Gillian Miller, Glasgow Royal Infirmary, Glasgow, UK; Gwen Edwards, University Hospital of Wales, Cardiff, UK; Haider Abbas, University Hospitals Leicester, Leicester, UK; Harris Siddique, Heart of England Hospital Trust, Birmingham, UK; Helen Lada, Imperial College Healthcare Trust, London, UK; Iain Wilson, Portsmouth University Hospitals, Portsmouth, UK; Ioannis Sarantitis, Royal Preston Hospital, Preston, UK; J. Bundred, Leeds University Hospitals, Leeds, UK; J. Gossage, Guy's and St Thomas' Hospital, London, UK; J. Sultan, Manchester University NHS Trust, Manchester, UK; J.A. Elliott, Beaumont Hospital, Bolton, UK and St James Hospital, Leeds, UK; Jane Dunn, Royal Sussex County Hospital, Brighton and Hove, UK; John Reynolds, St James Hospital, Leeds, UK; Jonathan Helbrow, University Hospitals Bristol, Bristol, UK; Jonathan Lessing, Leeds University Hospitals, Leeds, UK; Joy Murphy, Royal Sussex County Hospital, Brighton and Hove, UK; Judith Sayers, Leeds University Hospitals, Leeds, UK; Julie Walther, University Hospitals Bristol, Bristol, UK; Kaveetha Kandiah, University Hospitals Nottingham, Nottingham, UK; Katarina Chow, Guy's and St Thomas' Hospital Trust, London, UK; Kathryn Hogan, University Hospitals Bristol, Bristol, UK; Katie Roulston, University Hospital of Southampton, Southampton, UK; Khalid Akbari, University Hospital of Coventry and Warwick, Coventry, UK; Kish Pursnani, Royal Preston Hospital, Preston, UK; L. Brown, Edinburgh University, Edinburgh, UK; Lewis Gall, Glasgow Royal Infirmary, Glasgow, UK; Lewis Germain, Leeds University Hospitals, Leeds, UK; Liam Hyland, University Hospitals Nottingham, Nottingham, UK; Laura McGuinness, Imperial College Healthcare Trust, London, UK; Marie Kershaw, Heart of England Hospital Trust, Birmingham, UK; Mashal Ahmed, Heart of England Hospital Trust, Birmingham, UK; Matthaios Kapiris, Guy's and St Thomas' Hospital Trust, London, UK; Matthew Doe, University Hospitals Bristol, Bristol, UK; Mayar Aswad, University Hospitals Leicester, Leicester, UK; Mohammed Hamid, Heart of England Hospital Trust, Birmingham, UK; Mohammed Hoque, Heart of England Hospital Trust, Birmingham, UK; Mohan Hingorani, Hull Royal Infirmary, Hull, UK; Michael Glaysher, Portsmouth University Hospitals, Portsmouth, UK; Michael Hughes, Leeds University Hospitals, Leeds, UK; Nakul Viswanath, University Hospitals of Derby and Burton, Derby, UK; N. Blencowe, Leeds University Hospitals, Leeds, UK and University Hospitals Bristol, Bristol, UK; N.W. Han, Cambridge University Hospitals, Cambridge, UK; Nathalie Webber, University Hospitals Bristol, Bristol, UK; Nicholas Penney, Royal Surrey Hospital, Guildford, UK; Nicholas Maynard, Oxford University Hospitals, Oxford, UK; Nicholas Gomez, University Hospital of Wales, Cardiff, UK; Nila Tewari, University Hospital of Coventry and Warwick, Coventry, UK; Nitya Matcha, Guy's and St Thomas' Hospital Trust, London, UK; Nia Jackson, Morriston Hospital, Morriston, UK; Paul Williams, Leeds University Hospitals, Leeds, UK; P. Charlton, Oxford University Hospitals, Oxford, UK; P. Coe, Leeds University Hospital, Leeds, UK; P. Pucher, Guy's and St Thomas' Hospital Trust, London, UK and Portsmouth University Hospitals, Portsmouth, UK; P. Singh, Royal Surrey Hospital, Guildford, UK; Poppy Jones, Bradford Royal Infirmary, Bradford, UK; R. Samuel, Hull Royal Infirmary, Hull, UK; R. Goody, Imperial College Healthcare Trust, London, UK; R. Owen, Oxford University Hospitals, Oxford, UK; Raj Nijjar, Heart of England Hospital Trust, Birmingham, UK; Rania Mohammed, University Hospitals of Derby and Burton, Derby, UK; Reem Mahmood, Bradford Royal Infirmary, Bradford, UK; R.P.T. Evans, Queen Elizabeth Hospital Birmingham, Birmingham, UK; Rob Walker, University Hospital of Southampton, Southampton, UK; S. Markar, Imperial College Healthcare Trust, London, UK and Oxford University Hospitals, Oxford, UK; Said Alyacoubi, University Hospital of Southampton, Southampton, UK; Saleem Noormohamed, Heart of England Hospital Trust, Birmingham, UK; Sara Walker, Glasgow Royal Infirmary, Glasgow, UK; Sarah Gwynne, Morriston Hospital, Morriston, UK; Shameen Jaunoo, Royal Sussex County Hospital, Brighton and Hove, UK; Shiv Uppal, University Hospitals Leicester, Leicester, UK; Sinthuri Raveendran, Heart of England Hospital Trust, Birmingham, UK; Siobhan Chien, Glasgow Royal Infirmary, Glasgow, UK; Siona Growcott, University Hospitals Bristol, Bristol, UK; S.K. Kamarajah, Queen Elizabeth Hospital, Birmingham, Birmingham, UK; Sufyan Azam, Heart of England Hospital Trust, Birmingham, UK; Swetha Prathap, University Hospital of Wales, Cardiff, UK; T. Crosby, University Hospital of Wales, Cardiff, UK; T.J. Underwood, University Hospital Southampton, Southampton, UK; Tasia Aghadiuno, University Hospital of Southampton, Southampton, UK; Terence Lo, Hull Royal Infirmary, Hull, UK; Waleed Al-Khyatt, University Hospitals of Derby and Burton, Derby, UK; William Knight, Guy's and St Thomas' Hospital Trust, London, UK; Y.M. Goh, Oxford University Hospitals, Oxford, UK; Zubair Khanzada, University Hospitals of Derby and Burton, Derby, UK.

## Supplementary Material

zraf078_Supplementary_Data

## Data Availability

Data are not routinely available. The RadioRoux Steering Committee is open to applications for data access. Please contact the corresponding author for further information.

## References

[1] Kamangar F, Nasrollahzadeh D, Safiri S, Sepanlou SG, Fitzmaurice C, Ikuta KS et al The global, regional, and national burden of oesophageal cancer and its attributable risk factors in 195 countries and territories, 1990–2017: a systematic analysis for the global burden of disease study 2017. Lancet Gastroenterol Hepatol 2020;5:582–597 32246941 10.1016/S2468-1253(20)30007-8 PMC7232026

[2] Arnold M, Ferlay J, Van Berge Henegouwen MI, Soerjomataram I. Global burden of oesophageal and gastric cancer by histology and subsite in 2018. Gut 2020;69:1564–1571 32606208 10.1136/gutjnl-2020-321600

[3] Morgan E, Soerjomataram I, Gavin AT, Rutherford MJ, Gatenby P, Bardot A et al International trends in oesophageal cancer survival by histological subtype between 1995 and 2014. Gut 2021;70:234–242 32554620 10.1136/gutjnl-2020-321089

[4] Ando N, Kato H, Igaki H, Shinoda M, Ozawa S, Shimizu H et al A randomized trial comparing postoperative adjuvant chemotherapy with cisplatin and 5-fluorouracil *versus* preoperative chemotherapy for localized advanced squamous cell carcinoma of the thoracic esophagus (JCOG9907). Ann Surg Oncol 2012;19:68–74 21879261 10.1245/s10434-011-2049-9

[5] Yang H, Liu H, Chen Y, Zhu C, Fang W, Yu Z et al Neoadjuvant chemoradiotherapy followed by surgery *versus* surgery alone for locally advanced squamous cell carcinoma of the esophagus (NEOCRTEC5010): a phase III multicenter, randomized, open-label clinical trial. J Clin Oncol 2018;36:2796–2803 30089078 10.1200/JCO.2018.79.1483 PMC6145832

[6] Shapiro J, van Lanschot JJB, Hulshof MCCM, van Hagen P, van Berge Henegouwen MI, Wijnhoven BPL et al Neoadjuvant chemoradiotherapy plus surgery *versus* surgery alone for oesophageal or junctional cancer (CROSS): long-term results of a randomised controlled trial. Lancet Oncol 2015;16:1090–1098 26254683 10.1016/S1470-2045(15)00040-6

[7] Kelly RJ, Ajani JA, Kuzdzal J, Zander T, Van Cutsem E, Piessen G et al Adjuvant nivolumab in resected esophageal or gastroesophageal junction cancer. N Engl J Med 2021;384:1191–1203 33789008 10.1056/NEJMoa2032125

[8] Nilsson M, Olafsdottir H, Alexandersson von Döbeln G, Villegas F, Gagliardi G, Hellström M et al Neoadjuvant chemoradiotherapy and surgery for esophageal squamous cell carcinoma *versus* definitive chemoradiotherapy with salvage surgery as needed: the study protocol for the randomized controlled NEEDS trial. Front Oncol 2022;12:917961 35912196 10.3389/fonc.2022.917961 PMC9326032

[9] Deboever N, Jones CM, Yamashita K, Ajani JA, Hofstetter WL. Advances in diagnosis and management of cancer of the esophagus. BMJ 2024;385:e074962 38830686 10.1136/bmj-2023-074962

[10] Obermannová R, Alsina M, Cervantes A, Leong T, Lordick F, Nilsson M et al Oesophageal cancer: ESMO clinical practice guideline for diagnosis, treatment and follow-up. Ann Oncol 2022;33:992–1004 35914638 10.1016/j.annonc.2022.07.003

[11] Jones CM, Olsson Brown A, Dobeson C. NOTCH: the national oncology trainees collaborative for healthcare research. Clin Oncol 2020;32:632–635 10.1016/j.clon.2020.05.005 32487502

[12] von Elm E, Altman DG, Egger M, Pocock SJ, Gøtzsche PC, Vandenbroucke JP. The Strengthening The Reporting of OBservational studies in Epidemiology (STROBE) statement: guidelines for reporting observational studies. Int J Surg 2014;12:1495–149925046131

[13] Agha RA, Borrelli MR, Vella-Baldacchino M, Thavayogan R, Orgill DP. The STROCSS statement: Strengthening The Reporting Of Cohort Studies in Surgery. Int J Surg 2017;46:198–202 28890409 10.1016/j.ijsu.2017.08.586 PMC6040889

[14] National Guideline Alliance . Oesophago-gastric cancer: assessment and management in adults. In: *NICE Guideline NG83 Methods, Evidence and Recommendations*. National Guideline Alliance, 2018. https://www.nice.org.uk/guidance/ng83 (accessed 01 July 2024).

[15] Gurung I, Carr NJ, Horne J. The use of Royal College of Pathologists dataset guidelines in the reporting of oesophageal and gastric malignancies. J Pathol 2017;243:S27-S27

[16] National Cancer Institute . Common Terminology Criteria for Adverse Events (CTCAE) Version 4.0. NIH Publication, 2009. https://evs.nci.nih.gov/ftp1/CTCAE/CTCAE_4.03/Archive/CTCAE_4.0_2009-05-29_QuickReference_8.5x11.pdf (accessed 01 July 2024).

[17] Dindo D, Demartines N, Clavien PA. Classification of surgical complications: a new proposal with evaluation in a cohort of 6336 patients and results of a survey. Ann Surg 2004:240;205–213 15273542 10.1097/01.sla.0000133083.54934.ae PMC1360123

[18] Comparison of short-term outcomes from the international Oesophago-Gastric Anastomosis Audit (OGAA), the Esophagectomy Complications Consensus Group (ECCG), and the Dutch Upper gastrointestinal Cancer Audit (DUCA). BJS Open 2021;5:zrab010 35179183 10.1093/bjsopen/zrab010 PMC8140199

[19] Crosby T, Hurt CN, Falk S, Gollins S, Staffurth J, Ray R et al Long-term results and recurrence patterns from SCOPE-1: a phase II/III randomised trial of definitive chemoradiotherapy +/– cetuximab in oesophageal cancer. Br J Cancer 2017;116:709–716 28196063 10.1038/bjc.2017.21 PMC5355926

[20] Stahl M, Stuschke M, Lehmann N, Meyer HJ, Walz MK, Seeber S et al Chemoradiation with and without surgery in patients with locally advanced squamous cell carcinoma of the esophagus. J Clin Oncol 2005;23:2310–2317 15800321 10.1200/JCO.2005.00.034

[21] Bedenne L, Michel P, Bouché O, Milan C, Mariette C, Conroy T et al Chemoradiation followed by surgery compared with chemoradiation alone in squamous cancer of the esophagus: FFCD 9102. J Clin Oncol 2007;25:1160–1168 17401004 10.1200/JCO.2005.04.7118

[22] Van Hagen P, Hulshof MCCM, van Lanschot JJB, Steyerberg EW, Henegouwen MIB, Wijnhoven BPL et al Preoperative chemoradiotherapy for esophageal or junctional cancer. N Engl J Med 2012;366:2074–2084 22646630 10.1056/NEJMoa1112088

[23] Kamarajah SK, Evans RPT, Griffiths EA, Gossage JA, Pucher PH. Definitive chemoradiotherapy *versus* neoadjuvant chemoradiotherapy followed by radical surgery for locally advanced oesophageal squamous cell carcinoma: meta-analysis. BJS Open 2022;6:zrac125 36477836 10.1093/bjsopen/zrac125 PMC9728519

[24] Evans RPT, Kamarajah SK, Bundred JR, Siaw-Acheampong K, Nepogodiev D, Hodson J et al Rates of anastomotic complications and their management following esophagectomy: results of the Oesophago-Gastric Anastomosis Audit (OGAA). Ann Surg 2021;274:e850–e851 10.1097/SLA.0000000000004649 33630459

[25] Markar SR, Zaninotto G, Castoro C, Johar A, Lagergren P, Elliott JA et al LAsting Symptoms after Esophageal Resection (LASER). Ann Surg 2022;275:e392–e400 32404661 10.1097/SLA.0000000000003917

[26] Rees J, Hurt CN, Gollins S, Mukherjee S, Maughan T, Falk SJ et al Patient-reported outcomes during and after definitive chemoradiotherapy for oesophageal cancer. Br J Cancer 2015;113:603–610 26203761 10.1038/bjc.2015.258 PMC4647690

[27] van der Wilk BJ, Eyck BM, Doukas M, Spaander MCW, Schoon EJ, Krishnadath KK et al Residual disease after neoadjuvant chemoradiotherapy for oesophageal cancer: locations undetected by endoscopic biopsies in the preSANO trial. Br J Surg 2020;107:1791–1800 32757307 10.1002/bjs.11760 PMC7689829

[28] Eyck BM, van der Wilk BJ, Noordman BJ, Wijnhoven BPL, Lagarde SM, Hartgrink HH et al Updated protocol of the SANO trial: a stepped-wedge cluster randomised trial comparing surgery with active surveillance after neoadjuvant chemoradiotherapy for oesophageal cancer. Trials 2021;22:345 34001287 10.1186/s13063-021-05274-w PMC8127221

[29] van der Wilk BJ, Eyck BM, Wijnhoven BPL, Lagarde SM, Rosman C, Noordman BJ et al LBA75 neoadjuvant chemoradiotherapy followed by surgery *versus* active surveillance for oesophageal cancer (SANO-trial): a phase-III stepped-wedge cluster randomised trial. Ann Oncol 2023;34:S1317

[30] Chidambaram S, Owen R, Sgromo B, Chmura M, Kisiel A, Evans R et al Delayed surgical Intervention after Chemoradiotherapy in Esophageal cancer: (DICE) study. Ann Surg 2023;278:701–708 37477039 10.1097/SLA.0000000000006028

[31] Wang H, Tang H, Fang Y, Tan L, Yin J, Shen Y et al Morbidity and mortality of patients who underwent minimally invasive esophagectomy after neoadjuvant chemoradiotherapy *vs* neoadjuvant chemotherapy for locally advanced esophageal squamous cell carcinoma: a randomized clinical trial. JAMA Surg 2021;156:444–451 33729467 10.1001/jamasurg.2021.0133 PMC7970392

[32] Fan N, Wang Z, Zhou C, Bludau M, Contino G, Zhao Y et al Comparison of outcomes between neoadjuvant chemoradiotherapy and neoadjuvant chemotherapy in patients with locally advanced esophageal cancer: a network meta-analysis. EClinicalMedicine 2021;42:101183 34805809 10.1016/j.eclinm.2021.101183 PMC8585620

[33] Pasquali S, Yim G, Vohra RS, Mocellin S, Nyanhongo D, Marriott P et al Survival after neoadjuvant and adjuvant treatments compared to surgery alone for resectable esophageal carcinoma. Ann Surg 2017;265:481–491 27429017 10.1097/SLA.0000000000001905

[34] Kato K, Machida R, Ito Y, Daiko H, Ozawa S, Ogata T et al Doublet chemotherapy, triplet chemotherapy, or doublet chemotherapy combined with radiotherapy as neoadjuvant treatment for locally advanced oesophageal cancer (JCOG1109 NExT): a randomised, controlled, open-label, phase 3 trial. Lancet 2024;404:55–66 38876133 10.1016/S0140-6736(24)00745-1

