# Supplementary material for: Tolerability, toxicity, and outcomes following surgical and non-surgical approaches to the management of patients with locally advanced oesophageal squamous cell carcinoma: multicentre retrospective cohort study
Source: BJS Open. 2025 Sep 8;9(5):zraf078. doi: 10.1093/bjsopen/zraf078 (PMC12416563; doi:10.1093/bjsopen/zraf078)
Supplement: zraf078_Supplementary_Data [file zraf078_supplementary_data.docx]

Tolerability, toxicity and outcomes following surgical and non-surgical approaches to the management of patients with locally advanced oesophageal squamous cell carcinoma: multi-centre retrospective cohort study

The Radio-Roux Collaborative^*^

**Writing group listed in Appendix 1*

**Corresponding author:** Richard PT Evans, rptevans@doctors.org.uk

**Institute of Immunology and Immunotherapy, University of Birmingham.**

**Edgbaston. Birmingham**

**B15 2TT**

**Supplementary Appendix**

| Authorship list | Page 3 |
| --- | --- |

**Supplementary Figure and Tables**

| Supplementary Figure 1 – Centre Level Treatment Variation | Page 4 |
| --- | --- |
| Supplementary table 1 – CTCAE grade ≥3 Complications of oncological treatment | Page 5 |
| Supplementary table 2 Baseline characteristics of patients with oesophageal squamous cell carcinoma undergoing surgery | Page 6 |
| Supplementary table 3 Postoperative and pathological outcomes of patients with squamous cell carcinoma undergoing surgery | Page 7 |
| Supplementary table 4 Adjusted Cox Regression on long-term survival of patients with squamous cell carcinoma undergoing surgery | Page 8 |
| Supplementary table 5 Baseline characteristics of propensity matched cohort dCRT vs nCRT+surgery | Page 9 |

**Authorship List**

Writing Group

RPT Evans^12^, C Jones^8,24^, SK Kamarajah^12^, N Blencowe^8,^17, L Brown^25^, A Cockbain^7,^ P Coe^8^, T Crosby^22^, JA Elliott^1,16^, J Gossage^4^, NW Han^24^, S Markar^7,10^, R Owen^10^, P Pucher^4,11^, P Singh^14^, J Sultan^26^, TJ Underwood^22^, EA Griffiths^12^

Steering Group

RPT Evans^12^, C Jones^8,24^, SK Kamarajah^12^, N Blencowe^8,17^, L Brown^25^, J Bundred^8^, P Charlton^10^, A Cockbain^7^, P Coe^7^, T Crosby^22^, JA Elliott^1,16^, YM Goh^10^, R Goody^7^, J Gossage^4^, S Markar^7,10^, P Pucher^4,11^, G Radhakrishna^26^, R Samuel^6^, P Singh^14^, TJ Underwood^22^, EA Griffiths^12^

Collaborating Institutions

1. Beaumont Hospital
2. Bradford Royal Infirmary
3. Glasgow Royal Infirmary
4. Guys and St. Thomas’ Hospital Trust
5. Heart of England Hospital Trust
6. Hull Royal Infirmary
7. Imperial College Healthcare Trust
8. Leeds University Hospitals
9. Morriston Hospital
10. Oxford University Hospitals
11. Portsmouth University Hospitals
12. Queen Elizabeth Hospital Birmingham
13. Royal Preston Hospital
14. Royal Surrey Hospital
15. Royal Sussex County Hospital
16. St. James Hospital
17. University Hospitals Bristol
18. University Hospital of Coventry and Warwick
19. University Hospitals of Derby and Burton
20. University Hospitals Leicester
21. University Hospitals Nottingham
22. University Hospital Southampton
23. University Hospital of Wales

Additional Writing/Steering Group Institutions

1. Cambridge University Hospitals
2. Edinburgh University
3. Manchester University NHS Trust

Collaborators

Aaron Kisiel^12^, Aisha Anwer^5^,Ajay Mehta^8^, Alex Boddy^20^, Amy Irwin^12^, Annabel Lyles^8^, Ashley Poon-King^9^, Ashwin Krishnamoorthy^18^, Atia Khan^2^, Ben Grace^22^, Betsan Thomas^23^, Charles Rayner^14^, Claire Livings^4^, Craig Barrington^22^, Daniel Otter^8^, Daniel Newport^18^, David Fackrell^12^, Devesh Kaushal^18^, David McIntosh^3^, Ellhia Sudin^21^, Emma Upcurch^17^, Elena Theophilidou^21^, Fiona Crotty^16^, George Mori^2^, Geta Maharaj^9^, Gillian Miller^3^, Gwen Edwards^23^, Haider Abbas^20^, Harris Siddique5, Helen Lada^7^, Iain Wilson^11^, Ioannis Sarantitis^13^, Jane Dunn^15^, John Reynolds^16^, Jonathan Helbrow^17^, Jonathan Lessing^8^, Joy Murphy^15^, Judith Sayers^8^, Julie Walther^17^, Kaveetha Kandiah^21^, Katarina Chow^4^, Kathryn Hogan^17^, Katie Roulston^22^, Khalid Akbari^18^, Kish Pursnani^13^, William Knight^4^, Lewis Gall^3^, Lewis Germain^8^, Liam Hyland^21^, Laura McGuinness^7^, Marie Kershaw^5^, Mashal Ahmed^5^, Matthaios Kapiris^4^, Matthew Doe^17^, Mayar Aswad^20^, Mohammed Hamid^5^, Mohammed Hoque^5^, Mohan Hingorani^6^, Michael Glaysher^11^, Michael Hughes^8^, Nakul Viswanath^19^, Nathalie Webber^17^, Nicholas Penney^14^, Nicholas Maynard^10^, Nicholas Gomez^23^, Nila Tewari^18^, Nitya Matcha^4^, Nia Jackson^9^, Paul Williams^8^, Poppy Jones^2^, Raj Nijjar^5^, Rania Mohammed^19^, Reem Mahmood^2^, Rob Walker^22^, Said Alyacoubi^22^, Saleem Noormohamed^5^, Sara Walker^3^, Sarah Gwynne^9^, Shameen Jaunoo^15^, Shiv Uppal^20^, Sinthuri Raveendran^5^, Siobhan Chien^3^, Siona Growcott^17^, Sufyan Azam^5^, Swetha Prathap^23^, Tasia Aghadiuno^22^, Terence Lo^6^, Waleed Al-Khyatt^19^, Zubair Khanzada^19^

**Supplementary Figure 1 – Centre Level Treatment Variation**

| Centre | dCRT | nCRT | nCT | Surgery Only |
| --- | --- | --- | --- | --- |
| A | 10.5 | 63.2 | 5.3 | 21 |
| B | 11.1 |  | 88.9 |  |
| C | 21.5 | 36.6 | 18.3 | 23.6 |
| D | 29.7 | 32.4 | 21.6 | 16.3 |
| E | 37.1 | 35.7 | 10 | 17.2 |
| F | 41.7 | 8.3 | 50 |  |
| G | 43.8 | 37.5 | 12.5 | 6.2 |
| H | 44.3 | 50 | 2.9 | 2.8 |
| I | 50 | 10 | 33.3 | 6.7 |
| J | 51.6 | 22.6 | 6.5 | 19.3 |
| K | 53.1 | 16.7 | 25 | 5.2 |
| L | 60.4 | 10.2 | 22.4 | 7 |
| M | 60.9 | 8.7 | 18.8 | 11.6 |
| N | 61.7 | 4.3 | 30.4 | 3.6 |
| O | 65 | 1.9 | 27.2 | 5.9 |
| P | 69.8 |  | 23.8 | 6.4 |
| Q | 74.5 | 5.5 | 12.7 | 7.3 |
| R | 80.6 | 2.8 | 13.9 | 2.7 |
| S | 82.6 | 1.8 | 13.8 | 1.8 |
| T | 85.1 | 0.8 | 12.4 | 1.7 |
| U | 90.2 |  | 9.8 |  |
| V | 92.5 | 2.5 | 2.5 | 2.5 |
| W | 100 |  |  |  |

**Supplementary table 1 – CTCAE grade ≥3 Complications of oncological treatment**

| Complications | dCRT | nCRT | nCT | dCRT vs nCRT | dCRT vs nCT | nCRT vs nCT |
| --- | --- | --- | --- | --- | --- | --- |
|  | 923 | 218 | 286 | p values | p values | p values |
| **Hematological** | 5.68% | 6.81% | 5.23% | 0.23 | 0.39 | 0.22 |
| Anaemia (Hb<80g/L) | 1.44% | 0.85% | 0.33% | 0.21 | 0.08 | 0.22 |
| Febrile neutropenia | 4.33% | 5.53% | 4.25% | 0.19 | 0.48 | 0.25 |
| Thrombocytopenia (Platelets<50,000mm^3^) | 0.72% | 1.28% | 1.63% | 0.17 | 0.10 | 0.37 |
| Non-hematological | 29.31% | 20.85% | 18.63% | 0.002 | 0.001 | 0.26 |
| **Cardiac** | 1.44% | 0.85% | 1.31 | 0.12 | 0.44 | 0.30 |
| Myocardial infaction | 0.10% | 0.43% | 0.33% | 0.15 | 0.21 | 0.43 |
| New onset atrial/ventricular arrhythmia requiring urgent attention | 1.14% | 0.43% | 0.33% | 0.14 | 0.13 | 0.43 |
| **Dermatological** | 0.41% | 0.43% | 0.00% | 0.48 | na | na |
| Rash/acne covering 30% of the body surface area or requiring antibiotics | 0.41% | 0.43% | 0.00% | 0.48 | na | na |
| Hand foot syndrome, severe skin changes with pain | 0.00% | 0.00% | 0.00% | na | na | na |
| **Metabolic** | 2.68% | 0.85% | 0.33% | 0.03 | 0.01 | 0.22 |
| Hypomagnesaemia (Magnesium <0.4mmol/L) | 0.72% | 0.00% | 0.00% | na | na | na |
| Hypokalaemia (<3mmol/L) | 1.55% | 0.85% | 0.33% | 0.18 | 0.07 | 0.22 |
| Hypophosphatemia (causing hospitalisation) | 0.21% | 0.00% | 0.00% | na | na | na |
| Hyponatraemia (125-129 if symptomatic, <125 if asymptomatic) | 0.31% | 0.43% | 0.33% | 0.37 | 0.48 | 0.43 |
| **Pulmonary** | 6.19% | 5.11% | 1.96% | 0.24 | 0.01 | 0.03 |
| Dyspnoea-shortness of breath at rest or impacting quality of life | 0.62% | 0.43% | 0.33% | 0.35 | 0.30 | 0.43 |
| Pneumonia requiring antibiotics | 5.68% | 4.68% | 1.63% | 0.25 | 0.01 | 0.03 |
| **Constitutional(fatigue/weight loss)** | 3.51% | 4.26% | 2.61% | 0.27 | 0.25 | 0.15 |
| Fatigue not relieved by rest | 1.86% | 2.13% | 2.61% | 0.38 | 0.23 | 0.36 |
| Weight loss requiring tube feeding or TPN | 1.44% | 2.98% | 0.65% | 0.04 | 0.17 | 0.03 |
| **Gastrointestinal** | 14.14% | 11.49% | 7.84% | 0.12 | 0.01 | 0.08 |
| Diarrhoea (6 stools above baseline or hospitalisation indicated | 3.72% | 2.55% | 3.27% | 0.16 | 0.37 | 0.31 |
| Dysphagia, oesophagitis, tube feeding, TPN or hospitalisation indicated | 4.64% | 2.98% | 0.65% | 0.11 | 0.002 | 0.03 |
| Mucositis interfering with oral intake | 2.17 | 1.28% | 0.65% | 0.16 | 0.06 | 0.23 |
| Nausea vomiting, tube feeding, TPN or hospitalisation indicated. | 6.50% | 5.53% | 5.88% | 0.27 | 0.36 | 0.43 |
| Anorexia , tube feeding, TPN or hospitalisation indicated | 1.65% | 2.55% | 0.33% | 0.25 | 0.06 | 0.02 |
| Infection excluding pneumonia | 2.89% | 2.98% | 0.98% | 0.47 | 0.05 | 0.06 |
| **Vascular** | 2.37% | 2.55% | 2.29% | 0.43 | 0.47 | 0.42 |
| **Renal** | 2.37% | 1.28% | 1.63% | 0.12 | 0.25 | 0.37 |
| **Other** | 3.72% | 2.98% | 4.90% | 0.27 | 0.20 | 0.12 |

# Supplementary table 2 Baseline characteristics of patients with oesophageal squamous cell carcinoma undergoing surgery

|  |  | **dCRT** | **nCRT** | **nCT** | **Surgery Only** | **Total** | **p** |
| --- | --- | --- | --- | --- | --- | --- | --- |
| Total N (%) |  | 44 (8.2) | 171 (31.8) | 204 (38.0) | 118 (22.0) | 537 |  |
| Age | 18-44 | 2 (4.5) | 6 (3.5) | 2 (1.0) | 2 (1.7) | 12 (2.2) | <0.001 |
|  | 45-59 | 16 (36.4) | 55 (32.2) | 55 (27.0) | 23 (19.5) | 149 (27.7) |  |
|  | 60-79 | 24 (54.5) | 110 (64.3) | 142 (69.6) | 72 (61.0) | 348 (64.8) |  |
|  | >/=80 | 1 (2.3) | 0 (0.0) | 4 (2.0) | 21 (17.8) | 26 (4.8) |  |
|  | (Missing) | 1 (2.3) | 0 (0.0) | 1 (0.5) | 0 (0.0) | 2 (0.4) |  |
| Gender | Male | 18 (40.9) | 74 (43.3) | 86 (42.2) | 51 (43.2) | 229 (42.6) | 0.987 |
|  | Female | 26 (59.1) | 95 (55.6) | 115 (56.4) | 66 (55.9) | 302 (56.2) |  |
|  | Unknown | 0 (0.0) | 2 (1.2) | 3 (1.5) | 1 (0.8) | 6 (1.1) |  |
| ECOG Status | 0 | 19 (43.2) | 111 (64.9) | 100 (49.0) | 54 (45.8) | 284 (52.9) | 0.004 |
|  | 1 | 20 (45.5) | 54 (31.6) | 82 (40.2) | 49 (41.5) | 205 (38.2) |  |
|  | >/=2 | 5 (11.4) | 6 (3.5) | 22 (10.8) | 15 (12.7) | 48 (8.9) |  |
| Charlson Comorbidity Index | 0 | 38 (86.4) | 132 (77.2) | 157 (77.0) | 72 (61.0) | 399 (74.3) | 0.005 |
|  | 1-2 | 1 (2.3) | 29 (17.0) | 34 (16.7) | 33 (28.0) | 97 (18.1) |  |
|  | >/=3 | 1 (2.3) | 1 (0.6) | 2 (1.0) | 2 (1.7) | 6 (1.1) |  |
|  | (Missing) | 4 (9.1) | 9 (5.3) | 11 (5.4) | 11 (9.3) | 35 (6.5) |  |
| Tumor Grade | Well | 2 (4.5) | 6 (3.5) | 18 (8.8) | 17 (14.4) | 43 (8.0) | 0.005 |
|  | Moderate | 21 (47.7) | 79 (46.2) | 96 (47.1) | 45 (38.1) | 241 (44.9) |  |
|  | Poor | 16 (36.4) | 48 (28.1) | 60 (29.4) | 25 (21.2) | 149 (27.7) |  |
|  | Unknown | 5 (11.4) | 38 (22.2) | 30 (14.7) | 31 (26.3) | 104 (19.4) |  |
| Basaloid type SCC | No | 23 (52.3) | 121 (70.8) | 156 (76.5) | 81 (68.6) | 381 (70.9) | 0.060 |
|  | Yes | 3 (6.8) | 8 (4.7) | 7 (3.4) | 3 (2.5) | 21 (3.9) |  |
|  | Unknown | 18 (40.9) | 42 (24.6) | 41 (20.1) | 34 (28.8) | 135 (25.1) |  |
| Tumor Location | Middle 1/3 | 27 (61.4) | 66 (38.6) | 60 (29.4) | 50 (42.4) | 203 (37.8) | 0.001 |
|  | Lower 1/3 | 15 (34.1) | 90 (52.6) | 124 (60.8) | 51 (43.2) | 280 (52.1) |  |
|  | GOJ | 1 (2.3) | 9 (5.3) | 17 (8.3) | 9 (7.6) | 36 (6.7) |  |
|  | Unknown | 1 (2.3) | 6 (3.5) | 3 (1.5) | 8 (6.8) | 18 (3.4) |  |
| Tumor Length | Median (IQR) | 5.0 (3.1 to 5.0) | 5.0 (4.0 to 6.2) | 5.0 (4.0 to 6.0) | 3.0 (2.0 to 4.2) | 4.7 (3.0 to 6.0) | <0.001 |
| Staging CT | No | 3 (6.8) | 21 (12.3) | 7 (3.4) | 23 (19.5) | 54 (10.1) | <0.001 |
|  | Yes | 41 (93.2) | 150 (87.7) | 197 (96.6) | 95 (80.5) | 483 (89.9) |  |
| Staging PET | No | 2 (4.5) | 8 (4.7) | 5 (2.5) | 24 (20.3) | 39 (7.3) | <0.001 |
|  | Yes | 42 (95.5) | 163 (95.3) | 199 (97.5) | 94 (79.7) | 498 (92.7) |  |
| Staging MRI | No | 42 (95.5) | 167 (97.7) | 195 (95.6) | 117 (99.2) | 521 (97.0) | 0.270 |
|  | Yes | 2 (4.5) | 4 (2.3) | 9 (4.4) | 1 (0.8) | 16 (3.0) |  |
| Staging EUS | No | 16 (36.4) | 51 (29.8) | 68 (33.3) | 33 (28.0) | 168 (31.3) | 0.637 |
|  | Yes | 28 (63.6) | 120 (70.2) | 136 (66.7) | 85 (72.0) | 369 (68.7) |  |
| Staging EBUS | No | 44 (100.0) | 165 (96.5) | 200 (98.0) | 112 (94.9) | 521 (97.0) | 0.255 |
|  | Yes | 0 (0.0) | 6 (3.5) | 4 (2.0) | 6 (5.1) | 16 (3.0) |  |
| AJCC Clinical T Stage | T1a | 0 (0.0) | 1 (0.6) | 1 (0.5) | 25 (21.2) | 27 (5.0) | <0.001 |
|  | T1b | 2 (4.5) | 5 (2.9) | 9 (4.4) | 30 (25.4) | 46 (8.6) |  |
|  | T2 | 8 (18.2) | 30 (17.5) | 32 (15.7) | 35 (29.7) | 105 (19.6) |  |
|  | T3 | 33 (75.0) | 129 (75.4) | 150 (73.5) | 26 (22.0) | 338 (62.9) |  |
|  | T4a | 1 (2.3) | 6 (3.5) | 12 (5.9) | 2 (1.7) | 21 (3.9) |  |
|  | T4b | 0 (0.0) | 0 (0.0) | 0 (0.0) | 0 (0.0) | 0 (0.0) |  |
| AJCC Clinical N Stage | N0 | 19 (43.2) | 56 (32.7) | 75 (36.8) | 95 (80.5) | 245 (45.6) | <0.001 |
|  | N1 | 22 (50.0) | 84 (49.1) | 97 (47.5) | 16 (13.6) | 219 (40.8) |  |
|  | N2 | 3 (6.8) | 28 (16.4) | 27 (13.2) | 7 (5.9) | 65 (12.1) |  |
|  | N3 | 0 (0.0) | 3 (1.8) | 5 (2.5) | 0 (0.0) | 8 (1.5) |  |

# Supplementary table 3 Postoperative and pathological outcomes of patients with squamous cell carcinoma undergoing surgery

|  |  | **dCRT** | **nCRT** | **nCT** | **Surgery Only** | **Total** | **p** |
| --- | --- | --- | --- | --- | --- | --- | --- |
| Total N (%) |  | 44 (8.2) | 171 (31.8) | 204 (38.0) | 118 (22.0) | 537 |  |
| Overall complications | No | 35 (79.5) | 79 (46.2) | 107 (52.5) | 48 (40.7) | 269 (50.1) | <0.001 |
|  | Yes | 9 (20.5) | 92 (53.8) | 97 (47.5) | 70 (59.3) | 268 (49.9) |  |
| Major complications | No | 41 (93.2) | 123 (71.9) | 149 (73.0) | 82 (69.5) | 395 (73.6) | 0.019 |
|  | Yes | 3 (6.8) | 48 (28.1) | 55 (27.0) | 36 (30.5) | 142 (26.4) |  |
| Anastomotic Leak | None | 43 (97.7) | 147 (86.0) | 181 (88.7) | 107 (90.7) | 478 (89.0) | 0.623 |
|  | Type 1 | 1 (2.3) | 11 (6.4) | 9 (4.4) | 4 (3.4) | 25 (4.7) |  |
|  | Type 2 | 0 (0.0) | 6 (3.5) | 4 (2.0) | 3 (2.5) | 13 (2.4) |  |
|  | Type 3 | 0 (0.0) | 7 (4.1) | 10 (4.9) | 4 (3.4) | 21 (3.9) |  |
| Conduit Necrosis | No | 44 (100.0) | 164 (95.9) | 196 (96.1) | 114 (96.6) | 518 (96.5) | 0.601 |
|  | Yes | 0 (0.0) | 7 (4.1) | 8 (3.9) | 4 (3.4) | 19 (3.5) |  |
| Chyle Leak | No | 43 (97.7) | 164 (95.9) | 191 (93.6) | 112 (94.9) | 510 (95.0) | 0.618 |
|  | Yes | 1 (2.3) | 7 (4.1) | 13 (6.4) | 6 (5.1) | 27 (5.0) |  |
| Vocal Cord Paralysis | No | 44 (100.0) | 167 (97.7) | 197 (96.6) | 115 (97.5) | 523 (97.4) | 0.621 |
|  | Yes | 0 (0.0) | 4 (2.3) | 7 (3.4) | 3 (2.5) | 14 (2.6) |  |
| Pneumonia | No | 38 (86.4) | 138 (80.7) | 165 (80.9) | 78 (66.1) | 419 (78.0) | 0.004 |
|  | Yes | 6 (13.6) | 33 (19.3) | 39 (19.1) | 40 (33.9) | 118 (22.0) |  |
| Myocardial Infarctoin | No | 44 (100.0) | 171 (100.0) | 201 (98.5) | 117 (99.2) | 533 (99.3) | 0.379 |
|  | Yes | 0 (0.0) | 0 (0.0) | 3 (1.5) | 1 (0.8) | 4 (0.7) |  |
| pT Stage | pT0 | 2 (4.5) | 91 (53.2) | 27 (13.2) | 7 (5.9) | 127 (23.6) | <0.001 |
|  | pT1a | 2 (4.5) | 6 (3.5) | 9 (4.4) | 22 (18.6) | 39 (7.3) |  |
|  | pT1b | 6 (13.6) | 5 (2.9) | 17 (8.3) | 28 (23.7) | 56 (10.4) |  |
|  | pT2 | 4 (9.1) | 22 (12.9) | 27 (13.2) | 16 (13.6) | 69 (12.8) |  |
|  | pT3 | 18 (40.9) | 35 (20.5) | 104 (51.0) | 29 (24.6) | 186 (34.6) |  |
|  | pT4a | 1 (2.3) | 5 (2.9) | 10 (4.9) | 5 (4.2) | 21 (3.9) |  |
|  | Unknown | 11 (25.0) | 7 (4.1) | 10 (4.9) | 11 (9.3) | 39 (7.3) |  |
| pN Stage | N0 | 18 (40.9) | 126 (73.7) | 98 (48.0) | 79 (66.9) | 321 (59.8) | <0.001 |
|  | N1 | 12 (27.3) | 29 (17.0) | 56 (27.5) | 14 (11.9) | 111 (20.7) |  |
|  | N2 | 1 (2.3) | 8 (4.7) | 26 (12.7) | 11 (9.3) | 46 (8.6) |  |
|  | N3 | 2 (4.5) | 1 (0.6) | 13 (6.4) | 3 (2.5) | 19 (3.5) |  |
|  | Nx | 11 (25.0) | 7 (4.1) | 11 (5.4) | 11 (9.3) | 40 (7.4) |  |
| pM Stage | M0 | 28 (63.6) | 154 (90.1) | 180 (88.2) | 99 (83.9) | 461 (85.8) | <0.001 |
|  | M1 | 0 (0.0) | 0 (0.0) | 1 (0.5) | 3 (2.5) | 4 (0.7) |  |
|  | Mx | 16 (36.4) | 17 (9.9) | 23 (11.3) | 16 (13.6) | 72 (13.4) |  |
| Nodes Examined | Median (IQR) | 20.0 (14.5 to 26.0) | 23.0 (17.0 to 30.0) | 24.0 (18.0 to 31.5) | 22.0 (16.0 to 29.0) | 23.0 (17.0 to 30.5) | 0.181 |
| Nodes +ve | Median (IQR) | 0.0 (0.0 to 1.0) | 0.0 (0.0 to 1.0) | 1.0 (0.0 to 2.0) | 0.0 (0.0 to 0.8) | 0.0 (0.0 to 1.0) | <0.001 |
| Tumor Regression Grade | Unknown | 26 (59.1) | 23 (13.5) | 44 (21.6) | 118 (95.8) | 206 (38.4) | <0.001 |
|  | TRG 1 | 4 (9.1) | 84 (49.1) | 22 (10.8) | 0 (0.0) | 112 (20.9) |  |
|  | TRG 2 | 2 (4.5) | 26 (15.2) | 25 (12.3) | 0 (0.0) | 53 (9.9) |  |
|  | TRG 3 | 0 (0.0) | 24 (14.0) | 24 (11.8) | 0 (0.0) | 49 (9.1) |  |
|  | TRG 4 | 7 (15.9) | 8 (4.7) | 50 (24.5) | 0 (0.0) | 66 (12.3) |  |
|  | TRG 5 | 5 (11.4) | 6 (3.5) | 39 (19.1) | 0 (0.0) | 51 (9.5) |  |
| Proximal margin | R0 | 42 (95.5) | 170 (99.4) | 198 (97.1) | 115 (97.5) | 525 (97.8) | 0.293 |
|  | R1 | 2 (4.5) | 1 (0.6) | 6 (2.9) | 3 (2.5) | 12 (2.2) |  |
| Distal margin | R0 | 43 (97.7) | 171 (100.0) | 201 (98.5) | 117 (99.2) | 532 (99.1) | 0.374 |
|  | R1 | 1 (2.3) | 0 (0.0) | 3 (1.5) | 1 (0.8) | 5 (0.9) |  |
| CRM | R0 | 32 (72.7) | 157 (91.8) | 142 (69.6) | 95 (80.5) | 426 (79.3) | <0.001 |
|  | R1 | 12 (27.3) | 14 (8.2) | 62 (30.4) | 23 (19.5) | 111 (20.7) |  |
| LVI | No | 30 (68.2) | 148 (86.5) | 127 (62.3) | 94 (79.7) | 399 (74.3) | <0.001 |
|  | Yes | 14 (31.8) | 23 (13.5) | 77 (37.7) | 24 (20.3) | 138 (25.7) |  |
| PNI | No | 33 (75.0) | 155 (90.6) | 165 (80.9) | 106 (89.8) | 459 (85.5) | 0.005 |
|  | Yes | 11 (25.0) | 16 (9.4) | 39 (19.1) | 12 (10.2) | 78 (14.5) |  |
|  |  |  | 84 (49.1) | 24 (11.8) | 3 (2.5) | 112 (20.9) |  |
| Pathological Grade | Well | 1 (2.3) | 89 (52.0) | 39 (19.2) | 24 (20.3) | 153 (28.5) | <0.001 |
|  | Moderate | 19 (43.2) | 45 (26.3) | 93 (45.6) | 52 (44.1) | 209 (38.9) |  |
|  | Poor | 11 (25.0) | 22 (12.9) | 47 (23.0) | 23 (19.5) | 103 (19.2) |  |
|  | Unknown | 13 (29.5) | 15 (8.8) | 25 (12.3) | 19 (16.1) | 72 (13.4) |  |

# Supplementary table 4 Adjusted Cox Regression on long-term survival of patients with squamous cell carcinoma undergoing surgery

|  |  | **HR (univariable)** | **HR (multivariable)** |
| --- | --- | --- | --- |
| Age | 18-44 | - | - |
|  | 45-59 | 1.04 (0.42-2.59, p=0.929) | 1.87 (0.42-8.34, p=0.409) |
|  | 60-79 | 0.97 (0.40-2.36, p=0.945) | 1.87 (0.43-8.08, p=0.400) |
|  | >/=80 | 1.29 (0.47-3.55, p=0.622) | 2.08 (0.35-12.34, p=0.420) |
| Gender | Male | - | - |
|  | Female | 0.65 (0.51-0.85, p=0.001) | 0.47 (0.32-0.70, p<0.001) |
|  | Unknown | 1.05 (0.33-3.29, p=0.940) | 1.34 (0.17-10.35, p=0.782) |
| ECOG Status | 0 | - | - |
|  | 1 | 1.58 (1.21-2.06, p=0.001) | 1.15 (0.76-1.72, p=0.516) |
|  | >/=2 | 1.38 (0.88-2.17, p=0.164) | 1.21 (0.64-2.29, p=0.561) |
| Charlson Comorbidity Index | 0 | - | - |
|  | 1-2 | 1.16 (0.84-1.60, p=0.361) | 1.30 (0.82-2.07, p=0.270) |
|  | >/=3 | 1.05 (0.34-3.29, p=0.934) | 0.00 (0.00-Inf, p=0.993) |
| Tumor Grade | Well | - | - |
|  | Moderate | 2.10 (1.13-3.91, p=0.019) | 1.87 (0.82-4.23, p=0.135) |
|  | Poor | 2.32 (1.23-4.38, p=0.010) | 1.68 (0.73-3.86, p=0.221) |
|  | Unknown | 1.96 (1.02-3.77, p=0.045) | 0.86 (0.31-2.45, p=0.783) |
| Basaloid type SCC | No | - | - |
|  | Yes | 0.57 (0.25-1.28, p=0.171) | 0.31 (0.07-1.30, p=0.108) |
|  | Unknown | 0.99 (0.74-1.32, p=0.929) | 1.04 (0.63-1.72, p=0.872) |
| Tumor Location | Middle 1/3 | - | - |
|  | Lower 1/3 | 0.90 (0.68-1.18, p=0.438) | 0.56 (0.37-0.85, p=0.006) |
|  | GOJ | 1.05 (0.64-1.75, p=0.837) | 0.67 (0.34-1.33, p=0.252) |
|  | Unknown | 0.99 (0.48-2.04, p=0.984) | 0.46 (0.05-4.37, p=0.499) |
| Tumor Length (cm) | 1 | 1.02 (0.96-1.09, p=0.440) | 1.01 (0.94-1.09, p=0.759) |
|  | 1.5-<4 | 1.30 (1.24-1.21, p=0.040) | 1.10 (1.05-2.17, p=0.046) |
|  | 4-<6 | 2.37 (1.38-4.27, p=0.029) | 1.62 (0.65-7.42, p=0.326) |
|  | ≥6 | 3.17 (1.19-8.25, p=0.022) | 1.94 (0.49-8.80, p=0.327) |
| AJCC Clinical T Stage | T1a | - | - |
|  | T1b | 1.24 (0.50-3.03, p=0.642) | 0.21 (0.03-1.37, p=0.103) |
|  | T2 | 1.45 (0.64-3.28, p=0.368) | 1.19 (0.33-4.35, p=0.792) |
|  | T3 | 2.48 (1.16-5.28, p=0.019) | 1.73 (0.48-6.32, p=0.405) |
|  | T4a | 2.77 (1.09-7.05, p=0.032) | 1.74 (0.39-7.80, p=0.467) |
|  | T4b | NA (NA-NA, p=NA) | NA (NA-NA, p=NA) |
| AJCC Clinical N Stage | N0 | - | - |
|  | N1 | 0.99 (0.75-1.31, p=0.965) | 0.83 (0.54-1.28, p=0.404) |
|  | N2 | 1.31 (0.89-1.92, p=0.178) | 0.87 (0.47-1.59, p=0.642) |
|  | N3 | 1.41 (0.52-3.83, p=0.501) | 2.27 (0.43-12.03, p=0.335) |
| AJCC Clinical M Stage | M0 | - | - |
|  | M1 | 6.29 (0.87-45.22, p=0.068) | 3.06 (0.33-28.28, p=0.324) |
| Final Treatment | dCRT | - | - |
|  | nCRT | 0.52 (0.33-0.84, p=0.007) | 0.39 (0.20-0.78, p=0.008) |
|  | nCT | 0.84 (0.54-1.30, p=0.424) | 0.82 (0.45-1.49, p=0.519) |
|  | Surgery Only | 0.59 (0.36-0.95, p=0.032) | 0.87 (0.39-1.94, p=0.735) |

**Supplementary table 5 Baseline characteristics of propensity matched cohort dCRT vs nCRT+surgery**

|  |  | **dCRT** | **nCRT** | **Total** | **p** |
| --- | --- | --- | --- | --- | --- |
| Total N (%) |  | 394 (67.8) | 187 (32.2) | 581 |  |
| Age | 18-44 | 10 (2.5) | 5 (2.7) | 15 (2.6) | 0.972 |
|  | 45-59 | 98 (24.9) | 48 (25.7) | 146 (25.1) |  |
|  | 60-79 | 286 (72.6) | 134 (71.7) | 420 (72.3) |  |
| Gender | Female | 233 (59.1) | 107 (57.2) | 340 (58.5) | 0.861 |
|  | Male | 158 (40.1) | 78 (41.7) | 236 (40.6) |  |
|  | Unknown | 3 (0.8) | 2 (1.1) | 5 (0.9) |  |
| ECOG | 0 | 210 (53.3) | 108 (57.8) | 318 (54.7) | 0.399 |
|  | 1 | 155 (39.3) | 70 (37.4) | 225 (38.7) |  |
|  | >/=2 | 29 (7.4) | 9 (4.8) | 38 (6.5) |  |
| Charlson Comorbidity Index | 0 | 276 (70.1) | 135 (72.2) | 411 (70.7) | 0.957 |
|  | 1-2 | 87 (22.1) | 39 (20.9) | 126 (21.7) |  |
|  | >/=3 | 5 (1.3) | 2 (1.1) | 7 (1.2) |  |
|  | Missing | 26 (6.6) | 11 (5.9) | 37 (6.4) |  |
| Tumor Grade | Moderate | 191 (48.5) | 86 (46.0) | 277 (47.7) | 0.840 |
|  | Poor | 100 (25.4) | 48 (25.7) | 148 (25.5) |  |
|  | Unknown | 86 (21.8) | 42 (22.5) | 128 (22.0) |  |
|  | Well | 17 (4.3) | 11 (5.9) | 28 (4.8) |  |
| Basaloid type SCC | No | 287 (72.8) | 128 (68.4) | 415 (71.4) | 0.547 |
|  | Unknown | 93 (23.6) | 51 (27.3) | 144 (24.8) |  |
|  | Yes | 14 (3.6) | 8 (4.3) | 22 (3.8) |  |
| Tumor Location | GOJ | 16 (4.1) | 11 (5.9) | 27 (4.6) | 0.620 |
|  | Lower 1/3 | 178 (45.2) | 90 (48.1) | 268 (46.1) |  |
|  | Middle 1/3 | 187 (47.5) | 81 (43.3) | 268 (46.1) |  |
|  | Unknown | 13 (3.3) | 5 (2.7) | 18 (3.1) |  |
| Tumor Length | 1-<4 | 66 (16.8) | 25 (13.3) | 91 (15.6) | 0.945 |
|  | 4-<6 | 108 (27.4) | 43 (23.0) | 151 (26.0) |  |
|  | ≥6 | 73 (18.5) | 34 (18.1) | 107 (18.4) |  |
|  | Missing | 147 (37.3) | 85 (45.5) | 232 (39.9) |  |
| Staging CT | No | 22 (5.6) | 17 (9.1) | 39 (6.7) | 0.161 |
|  | Yes | 372 (94.4) | 170 (90.9) | 542 (93.3) |  |
| Staging PET | No | 13 (3.3) | 7 (3.7) | 20 (3.4) | 0.976 |
|  | Yes | 381 (96.7) | 180 (96.3) | 561 (96.6) |  |
| Staging MRI | No | 380 (96.4) | 181 (96.8) | 561 (96.6) | 1.000 |
|  | Yes | 14 (3.6) | 6 (3.2) | 20 (3.4) |  |
| Staging EUS | No | 145 (36.8) | 62 (33.2) | 207 (35.6) | 0.444 |
|  | Yes | 249 (63.2) | 125 (66.8) | 374 (64.4) |  |
| Staging EBUS | No | 381 (96.7) | 180 (96.3) | 561 (96.6) | 0.976 |
|  | Yes | 13 (3.3) | 7 (3.7) | 20 (3.4) |  |
| Staging Lap | No | 356 (90.4) | 155 (82.9) | 511 (88.0) | 0.014 |
|  | Yes | 38 (9.6) | 32 (17.1) | 70 (12.0) |  |
| AJCC Clinical T Stage | T1a | 3 (0.8) | 1 (0.5) | 4 (0.7) | 0.985 |
|  | T1b | 15 (3.8) | 7 (3.7) | 22 (3.8) |  |
|  | T2 | 60 (15.2) | 29 (15.5) | 89 (15.3) |  |
|  | T3 | 289 (73.4) | 140 (74.9) | 429 (73.8) |  |
|  | T4a | 25 (6.3) | 9 (4.8) | 34 (5.9) |  |
|  | T4b | 2 (0.5) | 1 (0.5) | 3 (0.5) |  |
| AJCC Clinical N Stage | N0 | 139 (35.3) | 66 (35.3) | 205 (35.3) | 0.975 |
|  | N1 | 191 (48.5) | 92 (49.2) | 283 (48.7) |  |
|  | N2 | 59 (15.0) | 26 (13.9) | 85 (14.6) |  |
|  | N3 | 5 (1.3) | 3 (1.6) | 8 (1.4) |  |
